# Deciphering pyritization-kerogenization gradient for fish soft-tissue preservation

**DOI:** 10.1038/s41598-017-01563-0

**Published:** 2017-05-03

**Authors:** Gabriel L. Osés, Setembrino Petri, Cibele G. Voltani, Gustavo M. E. M. Prado, Douglas Galante, Marcia A. Rizzutto, Isaac D. Rudnitzki, Evandro P. da Silva, Fabio Rodrigues, Elidiane C. Rangel, Paula A. Sucerquia, M. L. A. F. Pacheco

**Affiliations:** 1 Programa de Pós-graduação em Geoquímica e Geotectônica, Instituto de Geociências, Universidade de São Paulo - Rua do Lago 562, 05508080, Cidade Universitária, São Paulo-SP, Brazil; 2 Instituto de Geociências, Universidade de São Paulo - Rua do Lago 562, 05508080, Cidade Universitária, São Paulo-SP, Brazil; 3 Instituto de Geociências e Ciências Exatas, Universidade Estadual Paulista – Avenida 24A 1515, 13506900 Rio Claro-SP, Brazil; 4 Laboratório Nacional de Luz Síncrotron – Rua Giuseppe Maximo Scolfaro 10.000, 13083-970 Campinas-SP, Brazil; 5 Instituto de Física, Universidade de São Paulo - Rua do Matão 1371, 05508090, Cidade Universitária, São Paulo-SP, Brazil; 6 Departamento de Geologia, Universidade Federal de Ouro Preto – Morro do Cruzeiro s/n, 35400-000, Campus Morro do Cruzeiro, Ouro Preto-MG, Brazil; 7 Programa de Pós-Graduação em Química, Instituto de Química, Universidade de São Paulo – Avenida Prof. Lineu Prestes 748, 05508080, Cidade Universitária, São Paulo-SP, Brazil; 8 Departamento de Química Fundamental, Instituto de Química, Universidade de São Paulo – Avenida Prof. Lineu Prestes 748, 05508080, Cidade Universitária, São Paulo-SP, Brazil; 9 Laboratório de Plasmas Tecnológicos, Universidade Estadual Paulista – Avenida Três de Março 511, 18087-180 Sorocaba-SP, Brazil; 10 Departamento de Geologia, Universidade Federal de Pernambuco - Avenida Acadêmico Hélio Ramos s/n, 50740530, Cidade Universitária, Recife-PE, Brazil; 11 Departamento de Biologia, Universidade Federal de São Carlos – Rodovia João Leme dos Santos, Km 110, 18052780 Sorocaba-SP, Brazil

## Abstract

Soft-tissue preservation provides palaeobiological information that is otherwise lost during fossilization. In Brazil, the Early Cretaceous Santana Formation contains fish with integument, muscles, connective tissues, and eyes that are still preserved. Our study revealed that soft-tissues were pyritized or kerogenized in different microfacies, which yielded distinct preservation fidelities. Indeed, new data provided the first record of pyritized vertebrate muscles and eyes. We propose that the different taphonomic pathways were controlled by distinct sedimentation rates in two different microfacies. Through this process, carcasses deposited in each of these microfacies underwent different residence times in sulphate-reduction and methanogenesis zones, thus yielding pyritized or kerogenized soft-tissues, and a similar process has previously been suggested in studies of a late Ediacaran lagerstätte.

## Introduction

Exceptionally preserved fossils have palaeobiological novelties that are not often encountered elsewhere in the geological record. Housed in deposits known as Konservat-Lagerstätten^1^, investigation of fossil soft-tissues may provide unique insights into ancient biology and palaeoenvironmental conditions. Among these deposits, the Mesozoic rocks from the Santana Formation (Araripe Basin, northeast Brazil) stand out for their often exquisitely preserved and diverse fossil content, including plants, vertebrates (e.g., fishes, pterosaurs, dinosaurs, turtles and lizards), and invertebrates (e.g., insects, arachnids, and crustaceans)^2^. A remarkable case is the recent description of a fossilized heart and valves from fish, which provides insights into cardiac evolution^3^ and into the preservation of soft-tissues in other vertebrates^4, 5^.

The Crato Member (Supplementary Figure 1) is one of the most diverse palaeontological deposits from the Early Cretaceous period. Among the fish fossils from this area, *Dastible crandalli* (Supplementary Note 1) is the most abundant and has been found throughout the entire unit. Even though previous studies have briefly assessed the taphonomic aspects of Crato Member fossils^6^, several issues regarding taphonomic processes remain unresolved. Typically, the soft-tissues of these fossils have two distinct taphonomic modes: black carbonaceous compressions and orange iron oxyhydroxide, which occur in grey (GL) and beige (BL) limestones, respectively. Previous studies of insect preservation have interpreted this difference in preservation as a result of rock weathering^2^, but our evidence from fish tissues does not support this interpretation.

In this study, we hypothesized that fish have followed distinct preservation pathways according to palaeoenvironmental and/or microfacies variations. The results of our analyses confirmed that the fish were either pyritized or kerogenized in different sedimentary microfacies. We propose that the sedimentation rates varied in these microfacies, thus suggesting different carcass residence times either in sulphate-reduction (SR) or methanogenesis sedimentary microbial zones, which resulted in both different taphonomic pathways and preservation fidelities. Therefore, we suggest that the Crato Member fish fossilization occurred through a process similar to the kerogenization-pyritization gradient model for Neoproterozoic-Palaeozoic metazoan preservation^7, 8^.

## Results

**Figure 1:**
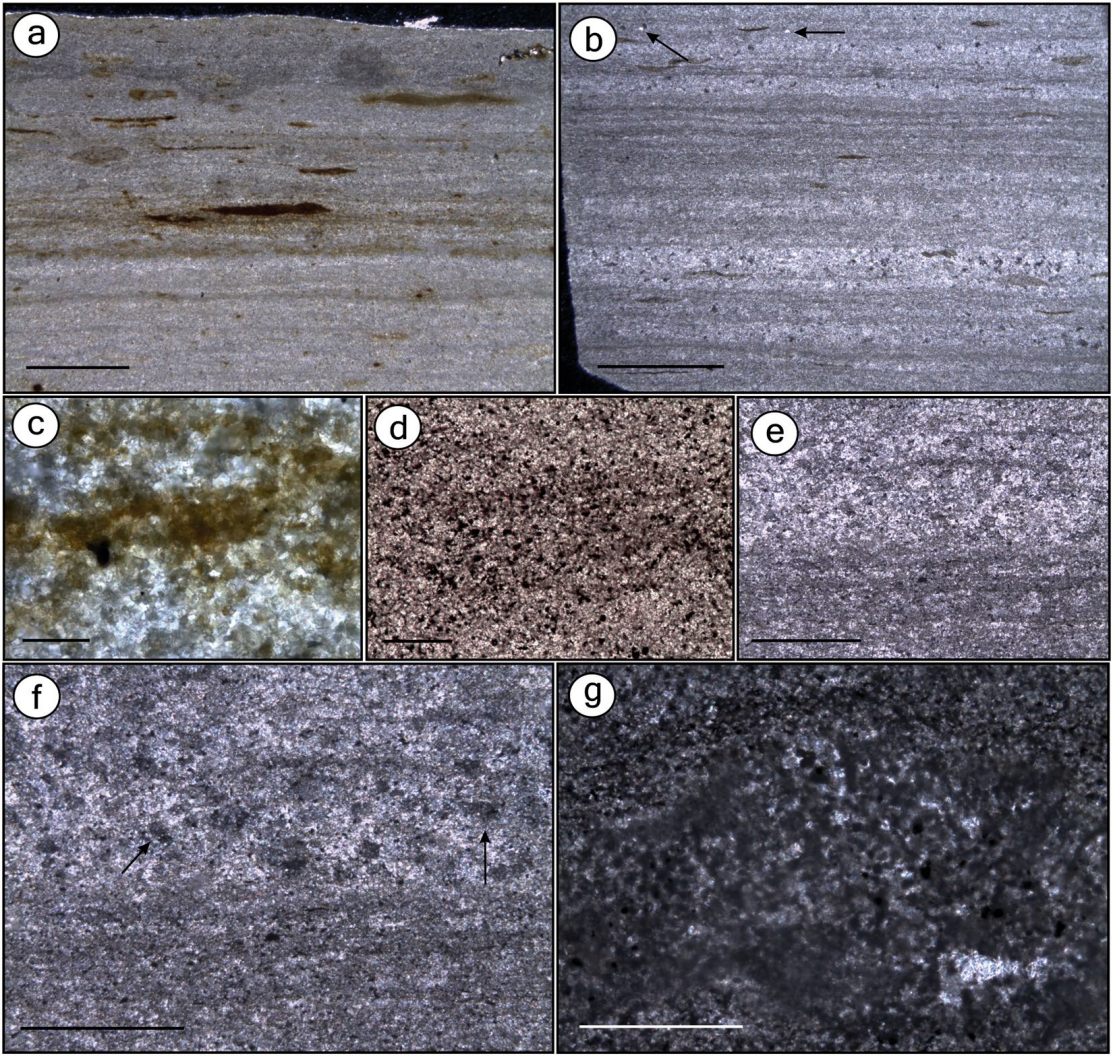
Thin sections of the beige limestone (BL) and the grey limestone (GL) microfacies. Thin sections GP/L 21 (**a**,**d**), GP/L 19 (**b**), GP/L 18 (**g**), GP/L 172 (**c**), and GP/L 16 (**e**,**f**). **(a**) BL is composed of thin laminasets of diffuse dark clay laminae (detail in **c**), interlaminated with pale pure microspar laminae. Elongated to round organic matter-rich dark lenses^11^ are indistinctly scattered. This microfacies is interpreted as the laminated limestone (LL) Sm5 microfacies^12^. (**b**) GL is composed of dark-grey undulated laminasets formed by thin laminae with fine blackish scattered material, likely clay/organic matter impurities^10, 12^ (detail in **d**). Laminasets are interlaminated with paler microspar-dominated laminae. Scattered non-oriented detrital quartz is indicated by arrow. GL microfacies is interpreted as Sm1, a clay-carbonate rythmite (CCR)^12^. Besides GL having significantly fewer dark lenses than BL, the clay/organic matter-rich laminasets are more frequent, regularly distributed, thicker and have more and closer-packed laminae. (**c)** Detail of BL dark laminae. (**d)** Detail of GL dark laminae, showing concentration of pyrite^10^. (**e)** Thin section of GL depicting microspar-dominated level (top) and clay-rich laminaset (bottom). (**f)** Image in (**e)** with crossed-nicols showing neomorphic sparry crystals (arrows). (**g)** GL clay-rich level with peloids. Scale bars: (**a)**– 1 mm; (**b)**– 2 mm; (**c)**– 0.02 mm; (**d)**– 0.1 mm; (**e)**– 0.5 mm; (**f)**– 0.5 mm; (**g)**– 0. 2 mm.

We compared soft-tissues from the fossil fish *Dastilbe crandalli* from the BL and GL microfacies. In Nova Olinda quarries, BL overlies GL^9, 10^, both differing mainly on siliciclastic influence. The BL are thinly laminated (ca. 0.5 mm) with dark clay interlaminated with pale pure microspar laminae (Fig. 1a,c) and have scattered dark, organic-rich lenses^11^. BL microfacies most probably corresponds to laminated limestones (LL)^12^. GL are composed of 1–3-mm-thick layers^12^ and have microfaults (Supplementary Figure 2). Undulated dark-grey pyrite-rich laminations^10^ exhibit fine black material (probably clay/organic impurities^10, 12^) and also peloids (Fig. 1b,d–g). Such laminations are interlaminated with paler microspar-dominated layers, including neomorphic sparry anhedral crystals^12^. We interpret the GL microfacies to be Sm1 microfacies or clay-carbonate rhythmite (CCR)^12^, which similarly to the GL, is composed of microfaults and millimetric plane-parallel and undulated laminae composed of intercalated clay and limestone.

### Fish from the BL microfacies

**Figure 2:**
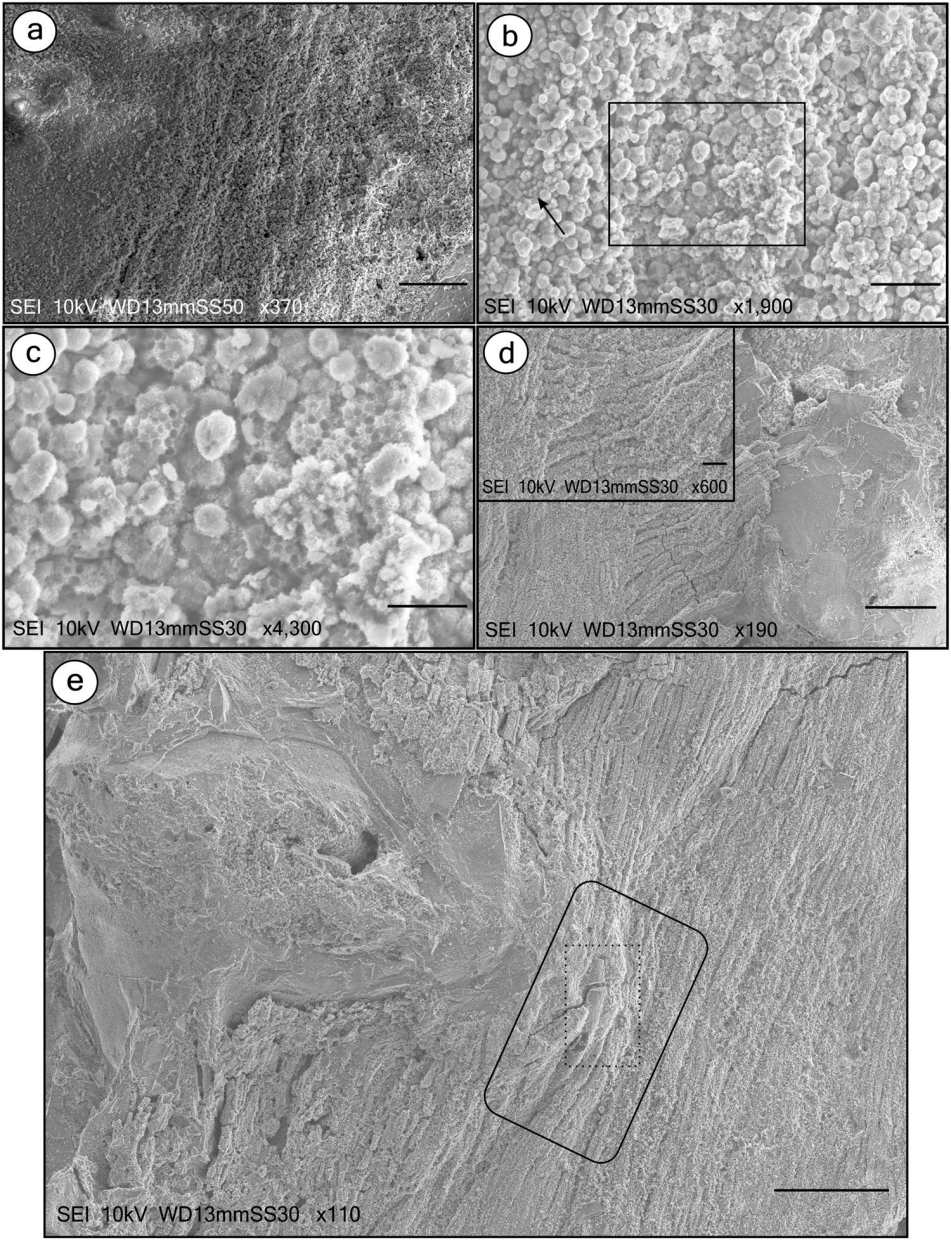
SEM of Crato Member BL fish soft-tissues and microfabrics. The images of the analyzed specimens and the localization of micrographs are depicted in Supplementary Figure 3. (**a**) depicts poorly-preserved muscle fibres, like diagonal ridges, at caudal fin base (Supplementary Figure 3). (**b**) shows that fibres are composed of Fe oxide/hydroxide (Supplementary Figure 5) sub-spherical to spherical grains with more than 1 µm (and occasionally less than this size, indicated by arrow), locally merged and covered by a “fuzzy” coating. The area highlighted in (**b)**, enlarged in (**c)**, depicts Fe oxide/hydroxide honeycomb-like texture (Supplementary Figure 5). (**d**), Micrograph showing how muscles are attached to dorsal fin base *via* tendons and the way these muscles connect to those sub-parallel to column (Supplementary Figure 3). Inset details these observations. Microfabric composition is depicted in Supplementary Figure 5. (**e**) Micrograph of fish anteroposterior axis (below dorsal fin; Supplementary Figure 3). Images reveal how muscles attach to vertebra, revealing preserved tendons (dashed rectangle), and muscles connected to tendons (big rectangle). Scale bars: (**a)**– 50 µm; (**b**)– 10 µm; (**c)**– 5 µm; (**d)**, (**e**)– 100 µm.

**Figure 3:**
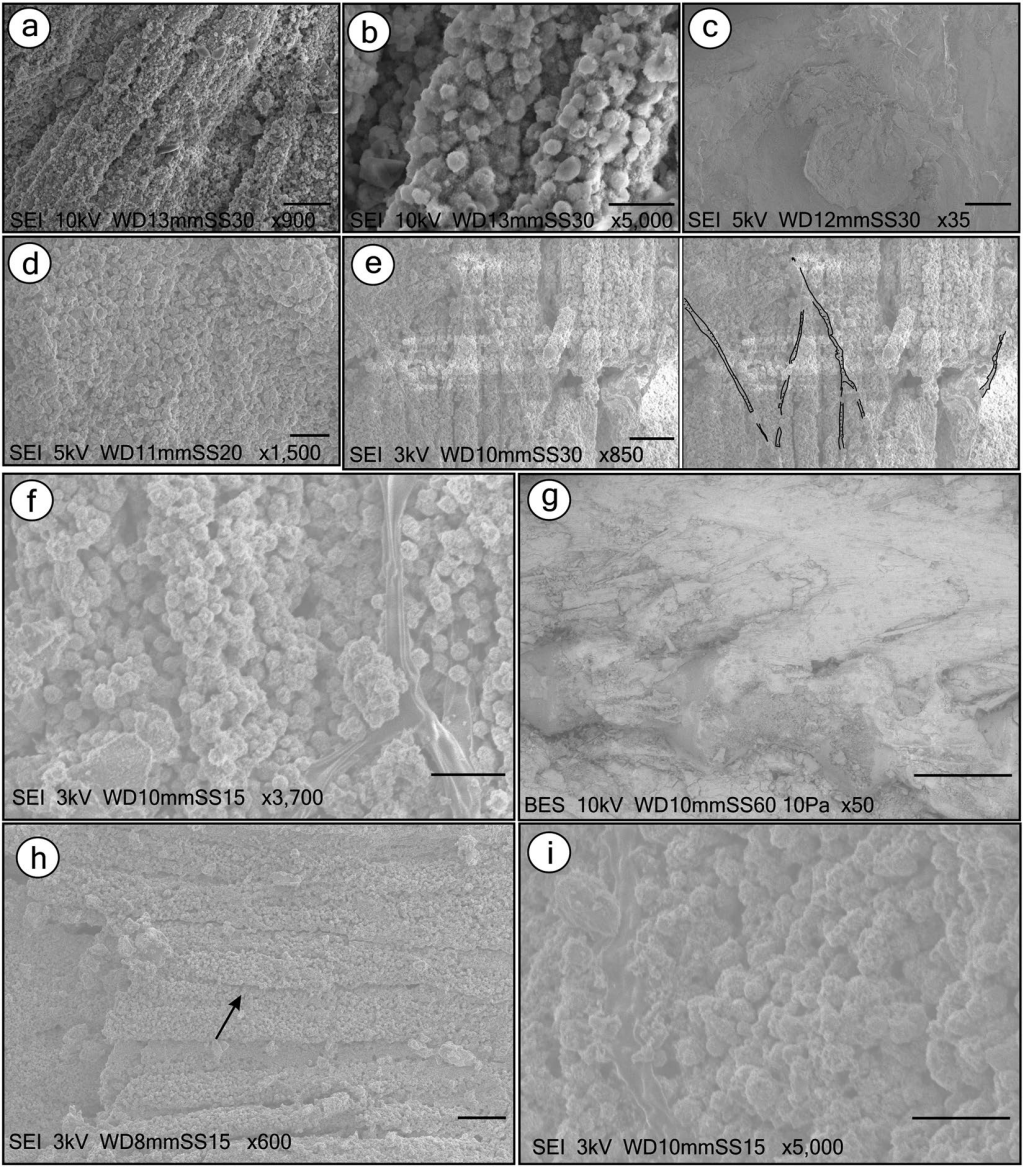
SEM of Crato Member BL fish soft-tissues, microfabrics and putative extracellular polymeric substances (EPS). The images of analyzed specimens and the localization of micrographs are depicted in Supplementary Figures 3, 4 and 6. (**a,b**) Micrographs of fish anteroposterior axis (below dorsal fin; Supplementary Figure 3) depicting fibre microfabric (Supplementary Figure 5). (**c**) depicts preserved eye (Supplementary Figure 6). (**d**) Shows eye microfabric. (**e**) (left) depicts fragmented and locally excavated muscle fibres subparallel to vertebral column. Gaps between fibres are occupied by wide, elongated, flat, soft-structures (sarcolemma), which also have orientations different from fibres. The same micrograph in the left is reproduced in the right with an interpretative drawing of the sarcolemma orientation. **(f**) Detail of sarcolemma (right). (**g**) shows myomeres with muscle fibres (Supplementary Figure 4). (**h**) Possible nucleus (arrow). (**i**), Putative EPS covering grains (Supplementary Figure 7) in left half of the figure. Scale bars: (**a**,**e**,**h)**– 20 µm; (**b**,**f**,**i)**– 5 µm; (**c**,**g)**– 500 µm; (**d)**– 10 µm.

In the most complete BL specimen, we analysed three distinct regions: caudal fin base, dorsal fin, and anteroposterior axis (Supplementary Figures 3 and 4). In the caudal fin base, the fibres possess scarcely visible margins and are nearly indistinguishable from each other (Fig. 2a). The fibres are composed of sub-spherical to spherical grains of iron oxide/hydroxide larger than 1 µm that are locally merged and covered by a “fuzzy” coating of submicroscopic crystals (Fig. 2b,c). These grains replaced muscles of the entire specimen. Similar, but smaller grains (<1 µm) occur in pores among larger grains (Fig. 2b). Iron oxide/hydroxide also may form a honeycomb-like texture characterized by subhedral to euhedral shaped hollows (Fig. 2c). We interpret that grains of up to 1 µm probably filled those spaces and were released after weathering oxidation^13^. The dorsal fin area exhibits muscles arranged in layers along the specimen’s depth, thus revealing how muscles are attached to the fin base and connected to muscles running along/around the column (Fig. 2d). Micrographs of the fish anteroposterior axis revealed muscular insertion into the vertebrae surface (including tendons attaching muscles to bones) and multiple stages of muscle decay around vertebra (Fig. 2e), and confirmed the fibre microfabric (Fig. 3a,b). These observations counter a previous analysis reporting rather indistinct fibres^6^. The eye area is also preserved by iron oxide/hydroxide microfabrics (Fig. 3c,d).

Patches of orange amorphous iron oxide/hydroxide material are commonly seen in BL fossils. These orange regions have 3D muscle fibres, as has been reported elsewhere^6^. The fibres in our samples may be fragmented and locally degraded, probably as a result of decay (Fig, 3e). Wide, elongated, flat, soft structures interpreted as sarcolemma (i.e., muscle cell membrane; Fig. 3e,f) occur between fibres. Despite being similar to putative EPS (extracellular polymeric substances) (below), the presence of these structures between fibres supports the hypothesis that they are sarcolemma, because a more random distribution would be present if they were EPS. Moreover, gaps between fibres also are present, which, along with occasional displacement of the sarcolemma in relation to such gaps^14^, may provide evidence for decay and dehydration/shrinkage of sarcolemma during fossilization. Some samples have fibres subparallel to the vertebral column that are arranged in myomeres connected to the dorsal column area (Fig. 3g,h), and some fibres possess putative cell nuclei (Fig. 3h).

In a specimen, we identified a ribbon-like smooth structure with a pliable aspect that shapes itself according to the microfabric relief underneath and resembles bacterially secreted EPS (Fig. 3i). Indeed, it is richer in carbon content than microfabric (Supplementary Figure 5). Similar structures have been found in Crato insects^15^.

**Figure 4:**
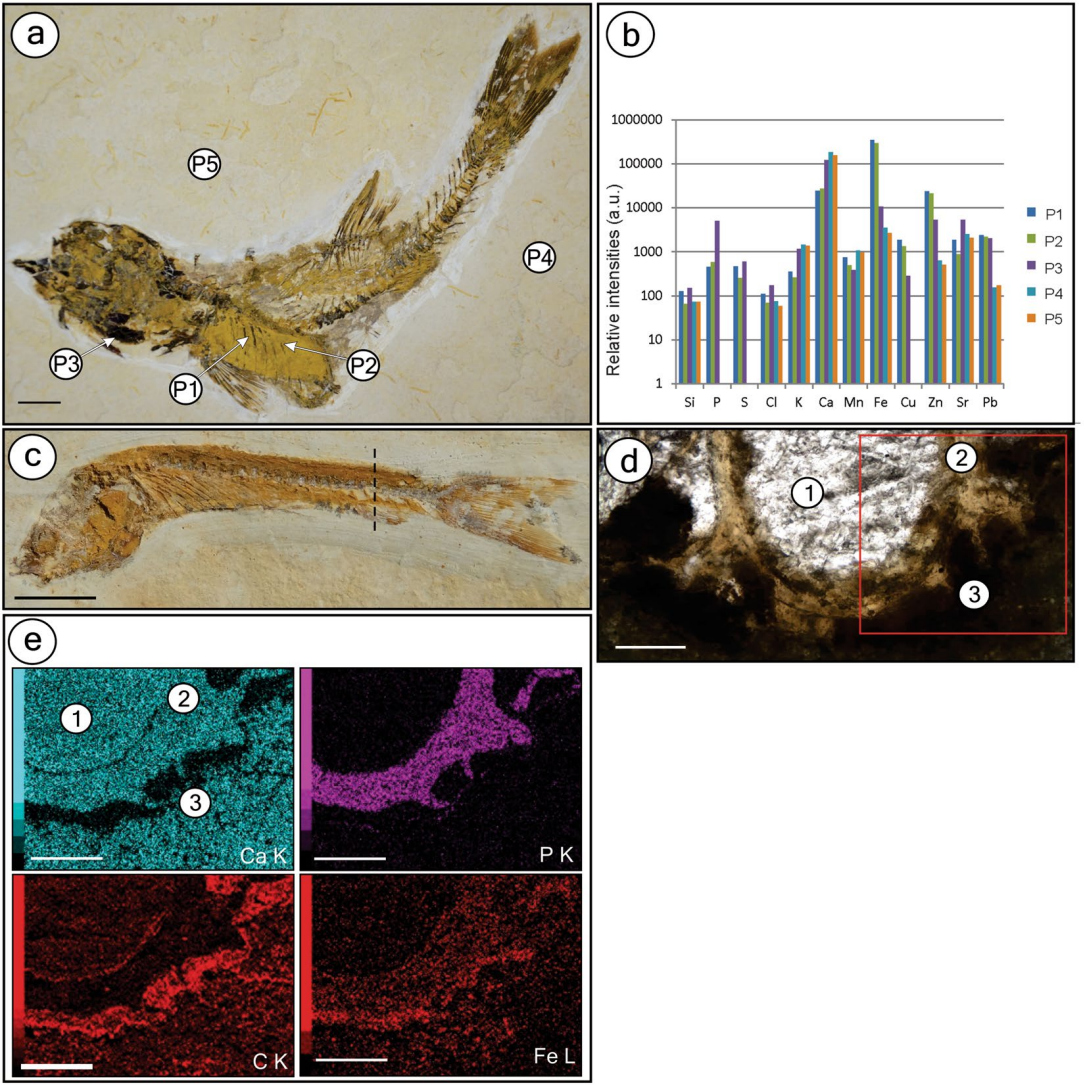
Geochemistry of the Crato Member fossil fish from BL. (**a**) Specimen GP/2E 9014 from BL with points (P1, P2 – decayed soft-tissue area; P3 – bones; P4, P5 – matrix) analyzed in (**b**). (**b**) EDXRF results of the selected points in fossil depicted in (**a**). (**c**) Fossil GP/2E 7781 g. The dashed line indicates the approximate direction of thin section in (**d**). (**d)** Thin section (GP/L 20) of specimen in **c** depicting calcite cement filling vertebrae (1), vertebrae (2), and soft-tissues (3) around vertebrae. The red square delimits the area analyzed in (**e)**. (**e)** Energy dispersive X-ray (EDS) maps of several elements distributed among the three main regions (1–3) in (**d)** Scale bars: (**a)**– 1 cm; (**c)**– 0.5 cm; (**d)**– 0.1 mm; (**e)**– 0.1 mm.

EDXRF analysis of fish from BL showed enhanced concentrations of phosphorus in bones from BL specimens compared with the rock matrix, whereas calcium is equally found in bones and in the carbonate matrix (Fig. 4a,b). The detection of a high abundance of phosphorus and calcium in soft-tissue areas is explained by the millimetric size of the X-ray beam of the system that was used, which measured bones scattered in these areas. We also measured lead, which correlated with the fossil and minor signals of sulphur and metals, such as iron, copper, and zinc, along soft-tissue regions (Fig. 4b).

EDS of one BL fish revealed original hydroxyapatite (Ca_5_(PO_4_)_3_(OH)) in bones, calcite-filled (CaCO_3_) bone cavities, and goethite (FeO(OH)) that replaced the soft-tissues surrounding vertebrae (Fig. 4c–e and Supplementary Figures 5 and 8). These results support the EDXRF data discussed above.

### Fish from GL microfacies

**Figure 5:**
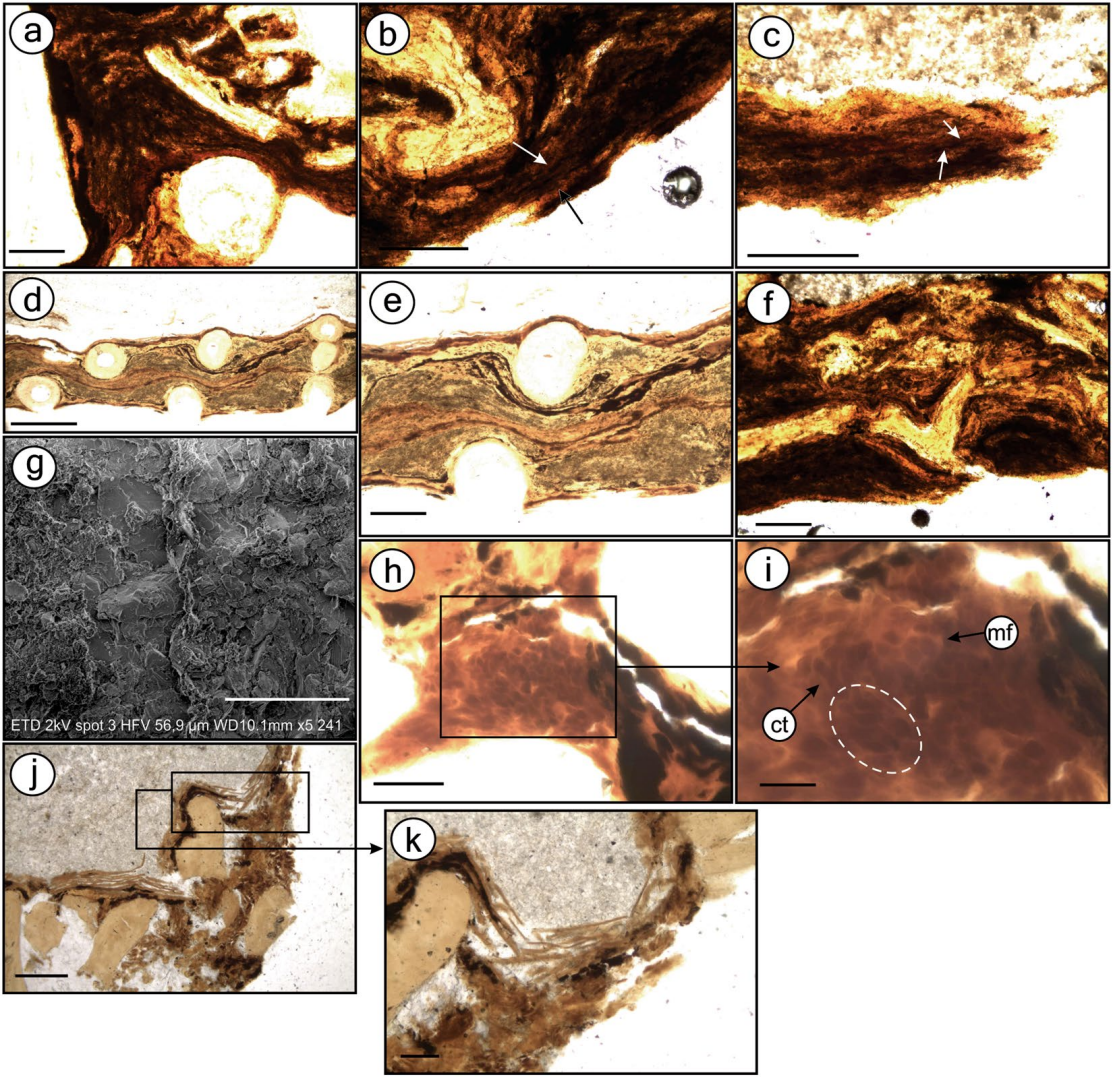
Thin section images and SEM of fish (Fig. 6a) with preserved carbonaceous soft-tissues. Thin sections GP/L 16 (**a**–**c**,**f**,**g**) and GP/L 17 (**d**,**e**,**h**–**k**). Dark opaque sinuous laminated, locally convolute muscle tissues (**a**–**c**), composed of alternating, differently coloured light/dark bands (**d**,**e**). Muscle tissue is composed of dark fibres (**b**,**c**; arrows). (**f**) Degraded soft-tissues and bones. (**g**) SEM micrograph of muscle fibre detail. (**h**,**i)** Muscle fibres (mf) in cross-section, depicting endomysium/perimysium-like connective tissues (ct) and muscle bundles (dashed ellipsis). (**j**,**k**) Scales are interlayered with soft-tissues (black) and calcite cement. Scale bars: (**a**–**c**,**e**,**f**,**j**,**k**)– 0.2 mm; (**d)**– 0.5 mm; (**g)**– 20 µm; (**h**)– 0.1 mm; (**i**)– 0.02 mm.

In carbonaceous fish, distinct cement types do occur (Supplementary Figure 2). Moreover, soft-tissues consist of a thick hard dark material that occurs consistently along the body. Almost every bone, in cross-section, is enveloped by dark opaque amorphous-to-sinuous fibrous material with alternating light/dark bands that are locally convoluted (Fig. 5a–c). Sometimes, black, beige, brown, and green bands alternate (Fig. 5d,e). We interpreted these features as preserved soft-tissue with muscle fibres (cells). In some regions, both soft-tissues and bones present a degraded aspect (Fig. 5f). Carbonaceous fibres are poorly preserved and have no discernible microfabrics (Fig. 5g).

Some regions of the anterior-posterior axis (particularly near a vertebrae) possess a vesicular texture formed by bodies of various shapes (Fig. 5h,i). They are locally oriented in a curved fashion that yields apparently round pockets. These bodies have been found to resemble muscle fibres in cross-section^16^. They are surrounded by a light matrix that resembles endomysium (i.e., collagenous connective tissue). The pockets can be interpreted as fascicles (i.e., muscle bundles) that are separated by a structure resembling perimysium, which is also a connective tissue for muscle fibres. Locally, the outer fish margin is outlined by broken scales interlayered with calcite and labile-tissue that we interpreted as being carbonaceous skin (Fig. 5j,k) (Supplementary Note 2). These aspects have rarely been documented in the fossil record. A detailed description of well-preserved micro-morphologies may facilitate exploration of new avenues for physiological research (e.g., insights into muscular functions) with evolutionary and palaeoecological implications^17^.

**Figure 6:**
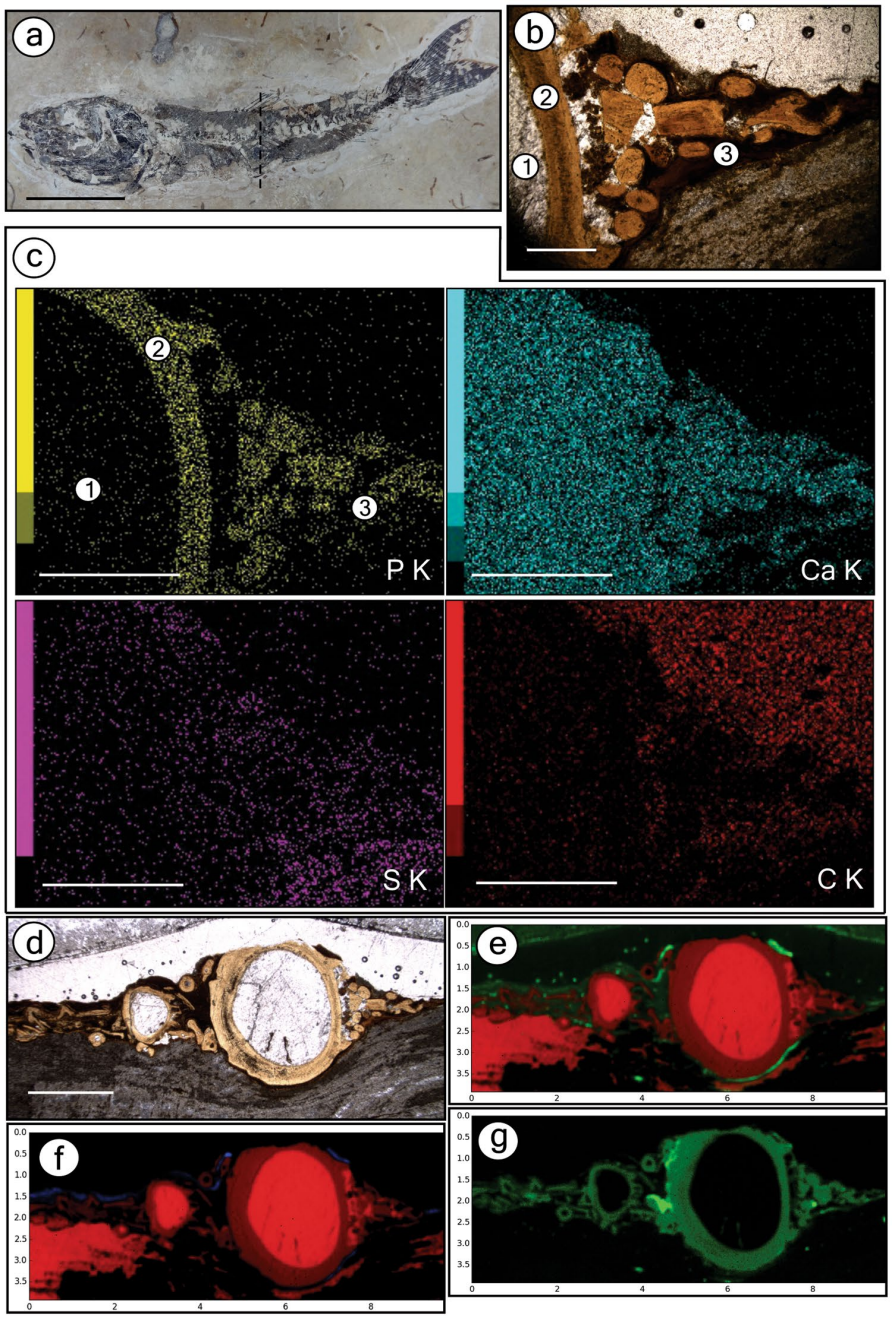
Geochemistry of the Crato Member fossil fish from GL. (**a**) Specimen GP/2E 9666 from grey limestones (GL). The approximate direction of thin section (in **b**) extraction is marked by dashed line. (**b)** Thin section (GP/L 16) of specimen in (**a**) showing calcite cement (1), vertebrae (2), and soft- tissues (3). (**c**) EDS maps of elemental distribution over areas (1–3) selected in (**b)**. (**d)** Area mapped by SR-µXRF of the thin section GP/L 16. (**e**–**g)** SR-µXRF maps. (**e)** Ca – red, Fe – green. (**f**) Ca – red, Cu – blue. (**g**) Zn. Colour brightness is proportional to element concentration, and both map horizontal and vertical scale axes are in mm. Elemental maps of all elements (except for Zn) are in Supplementary Figure 9. Scale bars: (**a)**– 1.5 cm; (**b)**– 0.5 mm; (**c)**– 1 mm; (**d)**– 2 mm.

The same bone composition from the BL was confirmed to be present in fish preserved in GL (Fig. 6a–e). In specimens from GL, iron and copper are more concentrated in soft-tissue regions (Fig. 6e,f), and zinc is more concentrated in bones (Fig. 6d,g). The soft-tissues from GL fish contain carbon (Fig. 6b,c) and sulphur (Supplementary Figure 9), thus revealing that they are mainly carbonaceous. Additionally, manganese is abundant in both the matrix and cement (Supplementary Figure 9).

## Discussion

### Pyritized Fish

In the BL specimens, the concentration of phosphorus together with calcium in bones indicated that the original hydroxyapatite is probably unaltered. Iron dominance in soft-tissues reflects the presence of iron oxides/hydroxides concentrated in these regions. Thus, the presence of sulphur can be explained by sulphate resulting from pyrite oxidation. Insects in BL are preserved as hematite/goethite replicas after pyrite^15^. Similarly, we interpret that fish soft-tissues in the BL were originally preserved by pyrite and later oxidized^6, 18^.

The microfabrics are mainly composed of iron oxide/hydroxide sub-spherical to spherical grains, which we interpret to be framboidal pyrite pseudomorphs^19^. This interpretation is enhanced by the occurrence of a honeycomb-like texture that resembles framboids. Regarding the hollow spaces in this honeycomb-like texture, which was probably formerly filled with microcrystals <1 µm, their morphology, size, and organization are consistent, thus supporting the interpretation that the honeycomb-like texture was originally a pyrite framboid^20, 21^.

Soft-tissue pyritization is inhomogeneous in the Crato Member fish. In the same specimen, some regions have framboids that are not associated with soft-tissues, whereas other regions have pyritized 3D muscles, particularly central trunk muscles surrounding the dorsal portion of the vertebral column, which is indeed the least decay-prone fish segment^22^. Myosepta (i.e., the collagen boundaries between myomeres) were not preserved in our samples. Moreover, some fish have fibres organized in myomeres, whereas others have non-organized muscle fibres, thus revealing a preservation gradient. Taphonomic experiments on amphioxus, lampreys, and fish have shown that the ventral myomere portion is lost before the dorsal one, and gaps develop between myomeres as they shrink after 6 decay-days^22^ (Supplementary Figure 4). Interestingly, some specimens with good muscle fibre preservation show evidence of integument rupture (Supplementary Figures 3 and 4; Supplementary Note 2), which should have supported sulphate-reducing bacteria (SRB) as well as sulphate and iron entry within carcasses, thereby promoting pyritization. Regarding eye preservation, the lens—which seems to be present in our specimen—is the least decay-prone eye structure, which may last more than 300 days under decay, as revealed by taphonomic experiments on lampreys^22^.

To our knowledge, despite of some reports of pyritized fish (fish from the Hunsrück Slate in Germany), this is the first description of pyritized vertebrate muscles and eyes in the fossil record. Surprisingly, Crato fish muscles are not phosphatized, as would be expected for vertebrate soft-tissue preservation (e.g., ref. 23). Because fish phosphatized soft-tissues are usually preserved by substrate microfabrics, phosphate crystals grow directly in decay sites, in a process requiring high availability of phosphate from external sources^14, 24^. Therefore, the lack of phosphatized tissues in the Crato Member fish may be explained by three main factors. First, BL had low C_org_ and associated phosphate accumulation^24, 25^, which controls mineralization^26^. Consequently, there was insufficient phosphate accumulation to inhibit calcite formation^24^. Second, phosphatization is enhanced at Ca-depleted continental basins^27^, which was not the case for the Crato palaeolake. Interestingly, Crato Member pterosaur phosphatized soft-tissues^4^ suggest that this process was taxon-controlled and localized in certain tissues, although a palaeoenvironmental influence cannot be ruled out. Future studies may test both hypotheses. Moreover, phosphatization occurs more rapidly than pyritization after burial^24^. Nevertheless, the Crato palaeolake was a euxinic (H_2_S-rich) basin^2^, and, as such, pyritization might have started even before burial, which would have made pyritization the very first widespread^28^ taphonomic mineralization process available for fish muscle and eye preservation.

Several conditions known to facilitate authigenic mineralization, such as anoxia^29^ and the lack of burrowers^30^, were present in the Crato palaeolake, owing to water column stratification^2^. Anoxia facilitates mineralization in the context of anaerobic respiration, and the absence of bioturbation prevents dilution of pore water electron acceptor concentrations, oxygenation of the substrate and the disruption of distinct geochemical gradients. Furthermore, microbial activity is thought to have influenced limestone deposition in the Crato palaeolake^11^. Therefore, the well-known role of microbial mats in controlling ion diffusion rates along the sediment^30, 31^ may also have influenced mineralization in the palaeolake. This ideal preservation scenario was ubiquitous in Precambrian siliciclastic marine settings (ref. 30; references therein), even though these environments were quite different from the Crato Member in depositional context. Nevertheless, as shown above, some factors may have created ideal conditions for soft-tissue preservation long after the closing of the Precambrian taphonomic window.

Soft-tissue preservation primarily depends on early diagenetic authigenic mineralization during anaerobic decay^25^ when geochemical gradients (e.g., pH, Eh)^26^ are created and lead to C_org_ mineralization^25^. In this process, bacteria utilize electron acceptors for anaerobic respiration (involving reduction reactions such as sulphate reduction and methanogenesis) and consumption (decay/oxidation) of organic matter (the electron donor in the redox reactions)^32^. These anaerobic respiration reactions ideally occur within several sedimentary metabolic zones (e.g., sulphate reduction, methanogenesis), and electron acceptors provide a higher free energy yield in shallower depths, thus resulting in mineral precipitation in geochemical zones (e.g., sulphidic or methanic)^33^. However, corresponding metabolic and geochemical zones do not necessarily match horizontally, and there may be an overlap among the levels of each zone^33^.

Among the mineralization processes, pyritization is of particular interest. Sulphate reduction bacteria (SRB) decay organic matter (i.e., energy source) by using SO_4_^2−^ as an electron acceptor in anaerobic respiration and commonly drive Fe^3+^ reduction to Fe^2+^ ^34^, thus leading to the mineralization of organic matter^28^. The precipitation of pyrite requires both reactive iron and sulphate environmental sources besides degradable organic matter^28^. We predict that a very fine balance among these factors created optimal conditions for fish labile-tissue pyritization in the Crato palaeolake. These three factors may have been controlled primarily by the depositional—e.g., the weathering-driven sources—and/or diagenetic processes discussed below. Indeed, the high productivity of the Crato palaeolake, which ultimately may reflect weathering nutrient contributions, has been proposed to have caused anoxia in the bottom waters^2^. The same argument has been used by Cui *et al*.^35^ to explain the conditions of the interplay between pyritization and biomineralization in Ediacaran oceans.

The sediments of the Crato palaeolake, a euxinic basin^2^, were dominantly calcareous, which are commonly iron-poor^28^. Therefore, pyrite genesis was iron-limited. The main source of sedimentary iron consists of Fe^3+^ oxyhydroxides derived from weathering^28^. Therefore, the Crato palaeoenvironment would have had an appreciable iron source, because its content was relatively significant compared to fossils, as supported by measurements of the matrix (Fig. 4b). Indeed, iron oxyhydroxides may have nourished the bottom waters of the palaeolake through pulses of freshwater^11^. Nevertheless, if iron quantities were still too low to yield carcass pyritization, this scenario would have been balanced by the low C_org_ of the BL^36^, because few decay sites (i.e., low widespread organic matter) would allow for focused bacterial sulphate reduction, sulphide fixation by iron at decay sites, and thus pyritization^25^ in fish.

Notably, pyritization occurred during lacustrine carbonate deposition in the Crato palaeolake, because such freshwater environments are usually sulphate-poor^24^, and pyritization is sulphate-limited^25, 28^. Thus, pyritization is commonly recorded in siliciclastic marine deposits^18, 31, 37, 38^ and in terrigenous lacustrine settings^39^, from oxic^30, 37, 38^ to dysoxic/anoxic^39^ water columns. However, considering the palaeolake water column O_2_ stratification^2, 11, 40^, anoxic bottom waters yielded an either shallow or absent sedimentary oxic zone with prevailing aerobic decay^30^. Therefore, bacterial iron/sulphate-reduction zones^34^ were shallow, thus facilitating iron/sulphate-enrichment^32^ and enhancing SR and pyritization^20^. Moreover, muscles and eyes (very labile tissues) probably provided intense SR, thus compensating for low sulphate input to SRB. Indeed, pristine C_org_ yields high sulphate-reduction rates^41^.

A total organic carbon (TOC) analysis of the Crato limestones revealed that the sediment was poor (<1%, although higher values have been recorded) in organic carbon^36^, possibly because of oxidation caused by bottom pulses of freshwater^11^ or by water stratification^36^. This TOC value may account for pyritization in BL, because low sedimentary C_org_ content widely explain pyritization^37, 38^. For carcasses being decomposed by SRB, hydrogen sulphide (H_2_S) is formed and must be readily fixed by Fe^2+^ for pyrite production to be concentrated in carcasses^20, 37, 38^. In contrast, sediments rich in C_org_ yield widespread pyritization, thus decreasing the contributions of sulphate and iron to fossil pyritization. Therefore, pyritization requires the sedimentary iron concentrations to exceed that of the sulphide produced by SRB in carcasses^20, 37, 38^.

Beyond the major palaeoenvironmental factors controlling pyritization, the fish carcasses themselves influenced pyrite production in the Crato palaeolake. Iron monosulphides nucleate because of sulphide supersaturation favoured by organic matter^19^. Moreover, depending on its nature, concentration and adsorption properties, organic matter can stabilize colloidal materials through particle aggregation (e.g., framboid formation) triggered by active C_org_ bridging, thus decreasing free energy^19^. Indeed, framboids have been widely associated with decay^42, 43^. Hence, putative EPS related to microfabrics (biofilm remains) may also have contributed to crystal binding of organic matter. Researchers have demonstrated the role of biofilms in developing proper structural and geochemical conditions for framboid development^44, 45^. Framboidal pyrite also replaced the tissue of Crato Member insects (interpreted as bacterially induced mineralization)^15^, and is also ubiquitous in other Konservat-Lagerstätten^18, 37, 46^. Moreover, the high abundance of copper, zinc, and lead (heavy metals commonly chelated by organic matter) in BL fish labile-tissues in comparison to the host rock (Fig. 4b) suggests the presence of microbial activity^47, 48^. These conditions may also reflect the incorporation of these metals into pyrite, as is favoured by high pyrite precipitation, especially in anoxic-sulphidic environments^49^ such as the Crato palaeolake. Indeed, framboids contribute to the mobilization of heavy metals, which concentrate zinc and lead, as compared with the concentrations in the host rock^50^. Together, the evidence in this study provides geochemical and mineralogical data, including putative EPS that suggest bacterially induced pyrite production.

There are some explanations for the distinct sizes of framboid pseudomorphs that replaced BL fish labile-tissues. The steps of framboidal pyrite precipitation^19^ involve (1) the nucleation and growth of iron monosulphides (FeS; mackinawite) from the reaction of Fe^2+^ and HS^−^, (2) the transition of mackinawite to greigite, (3) the aggregation of greigite microcrystals, and (4) the conversion of greigite to pyrite, although greigite is not always required^21^. The framboid sphericity plus microcrystal size/morphology—and consequently framboid size^13^—depend on the abovementioned steps. For example, in the process of monosulphide nucleation, each step—sulphate/Fe^3+^ reduction, crystal nucleation and crystal growth—starts when the previous step has finished. Thus, equally sized microcrystals are formed in a single framboid^19^. Otherwise, the overlap between nucleation and growth yields particles of different sizes, which lead to framboids of different sizes. Alternatively, the co-existence of distinct framboid sizes has been explained by the ‘particulation’ of original colloid droplets in a range of different final particle sizes^51^. It has also been argued that the variation of both framboid size and complexity is related to a continuous process of evolution from framboidal to euhedral pyrite, based chiefly on the occurrence of both textures with similar sizes and similarities between euhedral crystals and framboid microcrystals^13^.

We observed framboids with distinct sizes in addition to EPS replacing decayed labile-tissues (Fig. 3i). This evidence indicates that distinct sizes of framboid pseudomorphs replacing BL fish labile-tissues were the result of the geochemical background of a decay microenvironment. This size variation requires variable pyrite nucleation rates and iron/sulphate diffusion balance over time^7, 18, 21^, thus suggesting that iron and sulphate concentrations (besides pH values) vary over time, thus controlling framboid size (ref. 13; references therein). Whereas single pyrite crystals are formed under low Eh conditions, framboids precipitate under high Eh values, because the reaction of pyrite production is thermodynamically stable, thus yielding sulphide supersaturation and fast nucleation rates^21^. Initially pristine labile-tissues (fish muscles and eyes) yield high decay rates because of their high reactivity^52^, a key factor controlling pyrite production^28^. Consequently, sulphide supersaturation induces widespread nucleation sites, thus favouring framboid precipitation^21^. This influence of the decay potential of labile-tissues to pyrite habit (framboids or single euhedral crystals) has been demonstrated for Cambrian animals^18^ and is further supported by our data. The rapid nucleation of minute framboids (Fig. 2b) requires that ion diffusion to nucleation sites surpass the reaction speed^21^. In other words, iron and sulphate are used for creating more pyrite nuclei instead of fueling crystal growth. This process diminishes highly the availability of reactive organic matter for SRB over time, thus decreasing framboidal pyrite nucleation rates, and consequently, with a continuous influx of reactants, sulphide fuels crystal growth^7^, as suggested for the Eocene London Clay plants^53^ and Cambrian animals^18^. This ‘growth’ mechanism may account for the larger framboids that replaced the soft-tissues of the fish (Fig. 2c).

Preserved area extension and preservation fidelity are usually higher in smaller specimens, whereas larger specimens have only very localized poor soft-tissue preservation. This pattern was expected because smaller carcasses are more readily pyritized, because the required iron and sulphate content is lower, and fixation of sulphide by iron^20, 38^ is faster because of lower H_2_S production due to the already lower amount of decay-prone organic matter.

### Kerogenized Fish

We concluded that GL fish carbonaceous soft-tissues underwent kerogenization^54^, as evidenced by soft-tissue black colour, C composition^18^, lack of microfabrics, and isotropy at crossed nicols^54^. Moreover, micro-Raman data revealed D and G bands of disordered and graphitic carbon (Supplementary Figure 8), which are considered to be kerogen because the attribution of the carbonaceous material to a well-known biogenic source and the low thermal imprint in the Crato unit^40^ made other processes of disordered carbonaceous material genesis^55^—e.g., heating, metamorphism, and hydrothermalism—unlikely.

The two main mechanisms for resistant organic matter preservation^56^, degradation-recondensation and selective preservation, were not sufficient to explain the fish muscle preservation. However, muscle lipid-free aliphatic and ester-bound molecules can give rise to degradation-lasting polymeric components during diagenesis, which yields kerogen. Moreover, more labile-tissues undergo structural changes^57^, which might have accounted for muscle kerogenization.

Cement-filled vertebrae and inter-bone gaps have manganese concentrations, similar to those of the matrix, thus revealing that cement composition is either derived from matrix dissolution-reprecipitation during diagenesis or might reflect weathering-formed pyrolusite, as is frequently seen in Crato limestones^2^. Moreover, we interpret zinc-bone enrichment as being a remnant of original bone composition. Indeed, extant fish retain zinc in their bones^58^, thus possibly reflecting dietary availability, which also influences physiological processes^59^. Furthermore, iron and copper are concentrated in some soft-tissue regions in which high intensities of both elements are usually correlated (Fig. 6e,f). Because (1) evidence for mineralization has not been found in the kerogenized fish, and (2) zinc, iron and copper were highly restricted to either bones or muscles in our specimens, we considered whether these metals might have been incorporated during life, as seen in extant fish^58, 60, 61^. Indeed, we did not observe a distributional gradient of these metals between soft t-issues and their host rock (as was the case of pyritized fish), and thus the migration of heavy metals from the surrounding sediment in the GL microfacies was ruled out.

### Preservation Model

**Figure 7:**
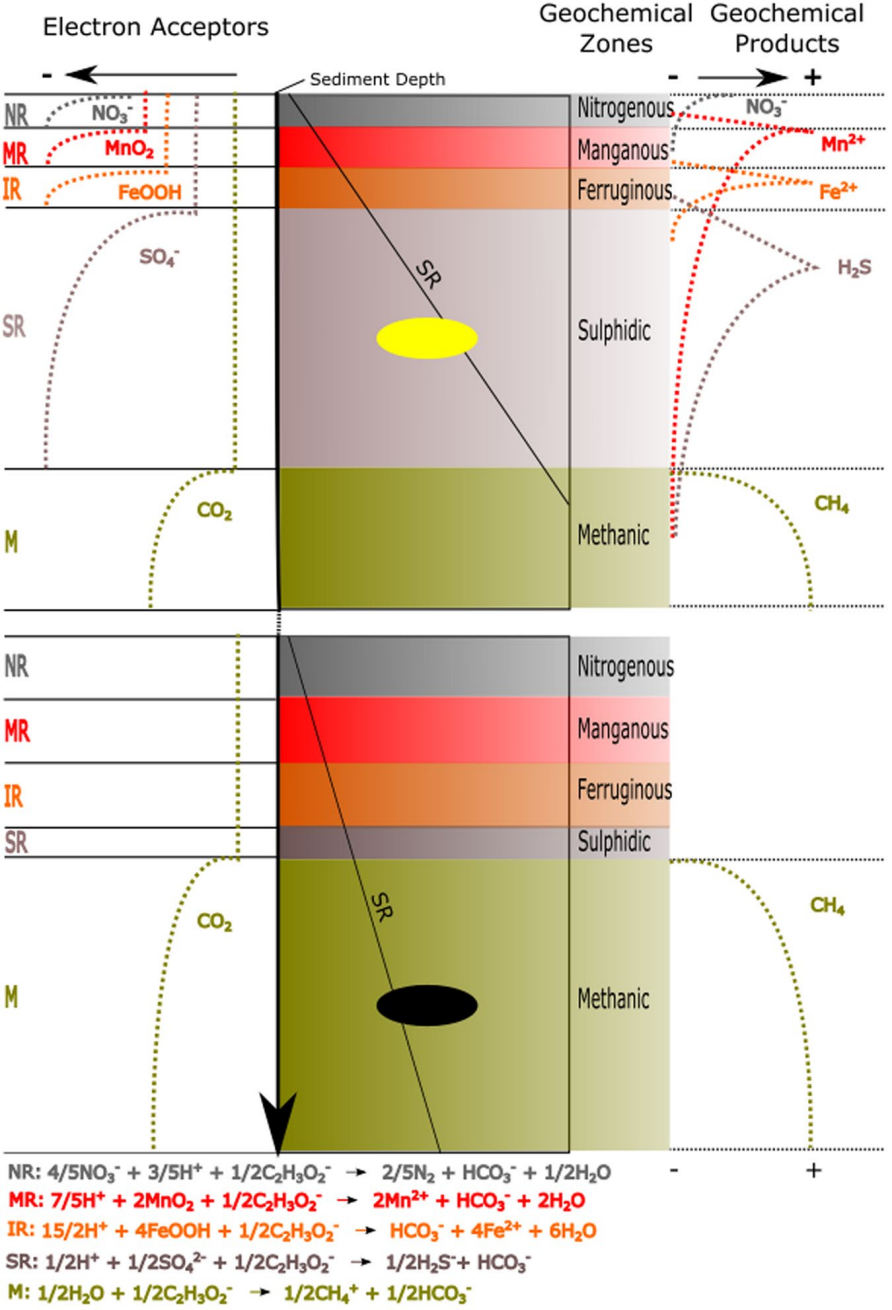
Taphonomic proposal for pyritization and kerogenization in the Crato Member. The upper and lower diagrams show bacterial respiration processes (NR – Nitrate Reduction, MR – Manganese Reduction, IR – Iron Reduction, SR – Sulphate Reduction, M - Methanogenesis) on the left. The correspondent reactions are seen at figure bottom. Electron acceptor curves used in these respiration processes are depicted, showing electron acceptor depletion from right to left (indicated by arrow at figure top). Sediment depth is represented by vertical arrow. Either pyritized (upper diagram) or kerogenized (lower diagram) fossils are represented by an ellipsis, located in the correspondent simplified sediment geochemical zone (sulphidic or methanic). Curves of respiration geochemical products are depicted on the right, showing increase from left to right. We propose that BL (upper diagram) has been deposited at lower sedimentation rates than GL (lower diagram) microfacies, as evidenced by greater terrigenous clay/organic matter content and peloid levels in GL. Variable sedimentation rates are explained by transgressive-regressive climatic cycles^12^. As a consequence, carcasses in BL have remained longer in the sulphidic zone, whereas carcasses in GL have both entered more rapidly and spent more time in the methanic zone, respectively yielding pyritized and kerogenized labile-tissues. In addition, variable cement contents in different carbonate microfacies^40^ plus clay in GL microfacies could have diminished sulphate percolation downwards, narrowing the sulphidic zone. The hypotheses we propose are based on refs 7 and 68. Sedimentation rate (SR) representation is based on ref. 7. Microbial respiration process zonation and reactions, geochemical zonation, plus electron acceptor and geochemical product curves are based on ref. 33.

In anaerobic bacterial respiration processes, particular electron acceptors are used in each respiration reaction (e.g., sulphate reduction and methane production), and the reactions ideally occur in a depth profile gradient within the sediment^33^ (Fig. 7). These respiration reactions yield geochemical products (e.g., sulphide and methane) that define sedimentary geochemical zones (e.g., sulphidic and methanic) that can overlap^33^ (Fig. 7). Schiffbauer *et al*.^7^ have proposed a model for predicting Neoproterozoic-Palaeozoic exceptional preservation by using a kerogenization-pyritization gradient that has further been examined by Cui *et al*.^35^, who have discussed the pyritization of Ediacaran fossils. This gradient is controlled by carcass residence time in distinct sediment anaerobic respiration microbial zones, especially the sulphate reduction zone (SRZ) and the methanogenesis zone^33, 62^. Residence time is controlled by sedimentation rate; hence, slow rates increase residence time in SRZ, yielding soft-tissue pyritization, whereas faster rates lead to shorter residence time in this zone, which favours kerogenization in the methanic zone^7^. We propose that this model can be extended to the Crato Member to explain pyritization in the BL and kerogenization in the GL microfacies (Fig. 7).

The Crato palaeolake had suitable conditions—including bottom water anoxia and absence of bioturbation^2^—for early diagenetic mineralization^29, 30^. The occurrence of neomorphic calcite in the limestone further supports the conclusion that diagenetic anaerobic conditions prevailed after fossilization, given that alkalinity drives neomorphism^35^. According to the rationale of Schiffbauer *et al*.^7^, the carcasses must have stayed in the SRZ for a period sufficiently long for pervasive pyritization to occur. This process would have been achieved through low sedimentation rates, which is supported by low C_org_ accumulation in the BL microfacies^36^ (our thin sections revealed fewer organic matter/clay-rich laminae than GL). Low burial rates would have yielded a shallow SRZ, thus favouring iron/sulphate concentration and consequently pervasive pyritization.

However, carcasses in the GL microfacies probably resided for a longer period in the methanogenesis zone because of increased burial rates (Fig. 7). According to petrographic evidence, the GL microfacies had higher organic matter/clay content (compared with BL), owing to higher terrigenous influence, as also potentially evidenced by its darker colour^63^. Moreover, round peloids do occur in specific layers, thus suggesting that they were deposited during sedimentary pulses^63^. Alternatively, their round shape might suggest *in situ* formation by microbial precipitation^63^. The more pronounced organic content in the host rock accounted for both pyrite scattered in the carbonate—which probably was also favoured by high iron quantities provided by terrigenous input^28^ during the GL deposition—and thus the lack of fish-pervasive pyritization (as explained in the above section). This rationale explains how carcasses passed through SRZ without being extensively pyritized^7^.

In addition to the presence of kerogen^7^, mineral paragenesis suggests the deposition of GL in the methanogenesis zone. There iss evidence for the presence of barite (BaSO_4_), such as the correlation intensity of barium and sulphur in a GL thin section (Fig. 6c; Supplementary Figure 9). Presumably, biogenic barite was dissolved in the sulphate-poor methanogenesis zone and provided barium for diagenetic barite precipitation (cf. ref. 64).

In fact, the variable terrigenous input was controlled by climatic conditions (humid *versus* arid)^11, 12^. Even considering that the clay contribution probably did not yield thick deposits at short intervals, the explanation above is sound. Indeed, it is known that slight depth changes (mm/cm-scale) are sufficient to change anaerobic zones in sediment^33, 65^. The faster sedimentation rates in GL may also have accounted for the lack of phosphatized fish soft-tissues in the Crato Member, because the carcasses would have been placed in a zone unfavourable for phosphatization.

In addition to the differential sedimentation rate hypothesis, Crato carbonate microfacies have varied cement contents^40^, which, together with clay in GL microfacies, possibly may have decreased downwards sulphate percolation and narrowed the SRZ^7^. The low microspar porosity might have enhanced this effect and also contributed to narrow other microbial zones, thereby eliminating the need for very high sediment amounts to move carcasses through these zones. This scenario would have led to premature sulphate exhaustion by SR, thereby hastening methanogenesis onset^62^ and preventing sulphide migration downwards and, consequently, further pyrite precipitation in the methanogenesis zone^33^.

In conclusion, a sedimentological-controlled preservation/fidelity gradient does exist for Crato Member fish, because pyritization has recorded 3D muscle tissues, sarcolemma, putative cell nuclei, tendons, and eyes, whereas kerogenization has yielded connective tissues, integument, and compressed/distorted muscle fibres. There is clearly a general trend for the mineralization of labile-tissues and the kerogenization of more recalcitrant structures^52^. Moreover, the former process has yielded greater preservation fidelity than the latter, probably because of the earlier onset of pyritization and to the higher degradability of labile-tissues, thereby leading to fast authigenic mineralization. Consequently, the types and the quality of preserved structures directly influence palaeobiological data retrieved from labile-tissues. Finally, we extended the taphonomic model of Schiffbauer *et al*.^7^—for Ediacaran-Cambrian early biomineralizing organisms from the Gaojiashan Formation (China) siliciclastic marine beds—to early Cretaceous fish from the Crato Member plattenkalk. Therefore, the taphonomic model for fish preservation in the Crato Member can be considered an analogue for the preservation of Ediacaran fossils in the Gaojiashan Formation, thus potentially confirming the wide applicability of the Schiffbauer pyritization-kerogenization model beyond the terminal Ediacaran to wherever these mixed taphonomic modes are found.

## Material and Methods

We have analyzed samples and thin sections (Supplementary Table) of the fossil fish *Dastilbe crandalli*. Although the exact provenance of this material was not specified after collection, it belongs to the Crato Member (Santana Formation, Araripe Basin, north-east Brazil) and is deposited in the Palaeontological Scientific Collection of the Institute of Geosciences of the University of São Paulo (USP).

Thin sections cross-cutting rock lamination and fossils were prepared. While 30 µm-thick thin sections with cover slips were used for rock and soft-tissue description, thin sections, thicker than 30 µm (ca. 50 µm) devoid of cover slips were employed in geochemical and scanning electron microscopy analyzes. The latter present more rock volume than usual 30 µm thin sections, which yields enhanced signal for mapping^6^. We took images from thin sections in transmitted light mode.

Energy dispersive X-ray fluorescence (EDXRF) analyzes were performed in the Institute of Physics of USP (IF-USP). We used a portable equipment setup that consists of a mini Amptek X-ray tube of Ag anode and a silicon drift detector (SDD), with 125 eV FWHM for the 5.9 keV line of Mn. The measurements were carried out with 30 kV voltage and 5 µA current and with an excitation/detection time of 100 s. Data were then processed in the software WinQXAS and Microsoft Excel^®^. The SR-µXRF measurements were made at the XRF beamline of the Brazilian Synchrotron Light Laboratory (LNLS/CNPEM)^66^ in micro-beam mode, using polychromatic excitation, filtered with Fe and Al foils. A KB focusing system was employed to achieve a beam size of approximately 12 × 25 μm. Mappings were made with 20 μm steps. The accumulation time was of 0.2 s per point, and acquisition was made in fly-scan mode. Control spectra were collected in both the glass slide and the glue used for thin-section preparation, as well as in untreated fossils and their host rock to ensure that the fossils’ real data were being measured in thin sections. All data were treated using the PyMca software^67^ for the creation of elemental concentration semi-quantitative maps.

Soft-tissue micro-investigation was carried out by Scanning Electron Microscopy (SEM), using a JEOL JSM-6010 LA microscope and a FEI Quanta 650 FEG microscope at the Brazilian Nanotechnology Laboratory (LNNano) and at the Laboratory of Technological Plasmas (LaPTec) of the São Paulo State University (UNESP). In order to avoid surface charging during micrograph acquisition, some samples were coated with a thin layer of gold-palladium in a DESK-V HP Cold Etch/Sputter system. We used an X-ray Dry SD Hyper (EX-94410T1L11) detector for Energy-Dispersive X-ray Spectroscopy (EDS) microanalysis. It yielded point spectra and maps of samples and thin sections, enabling the evaluation of elemental distribution among distinct fossil regions. We avoided performing EDS measurements of coated samples.

Raman spectroscopy was performed both at the Research Unity in Astrobiology, Laboratory of Astrobiology (NAP/Astrobio-USP) and at the Laboratory of Molecular Spectroscopy (LEM) of the Institute of Chemistry of USP. Raman data were collected in micro mode with two different Renishaw inVia micro-Raman systems coupled to confocal light microscopes, one with 532 nm excitation laser line, and another with 785 nm excitation line. Equipment was set to provide spectral resolutions of about 4 cm^−1^, being calibrated by the Si band at 520.7 cm^−1^. We used a 20X objective lens, exposure times of 10 s and 15 s, and laser powers of 0.05% and 5%. Point spectra were produced using both Renishaw WiRE^TM^ 4.1 and Origin^*®*^8 software. Data were interpreted using the RRUFF database.

## Electronic supplementary material

Supplementary Information
