# Supplementary material for: Deciphering pyritization-kerogenization gradient for fish soft-tissue preservation
Source: Sci Rep. 2017 May 3;7:1468. doi: 10.1038/s41598-017-01563-0 (PMC5431149; doi:10.1038/s41598-017-01563-0)
Supplement: Supplementary file 1 — Supplementary Information [file 41598_2017_1563_MOESM1_ESM.pdf]

Deciphering pyritization-kerogenization gradient for fish soft-tissue preservation

Gabriel L. Osés*[1], Setembrino Petri[2], Cibele G. Voltani[3], Gustavo M. E. M. Prado[1], Douglas Galante[4], Marcia A. Rizzutto[5], Isaac D. Rudnitzki[6], Evandro P. da Silva[7], Fabio Rodrigues[8], Elidiane C. Rangel[9], Paula A. Sucerquia[10], M. L. A. F. Pacheco[11]

[1]Programa de Pós-graduação em Geoquímica e Geotectônica, Instituto de Geociências, Universidade de São Paulo - Rua do Lago 562, 05508080, Cidade Universitária, São Paulo-SP, Brazil - E-mail: gabriel.oses@usp.br, gustavo.dino@gmail.com.

[2]Instituto de Geociências, Universidade de São Paulo - Rua do Lago 562, 05508080, Cidade Universitária, São Paulo-SP, Brazil - E-mail: spetri@usp.br.

[3]Instituto de Geociências e Ciências Exatas, Universidade Estadual Paulista – Avenida 24A 1515, 13506900, Rio Claro-SP, Brazil - E-mail: voltani@rc.unesp.br.

[4]Laboratório Nacional de Luz Síncrotron – Rua Giuseppe Maximo Scolfaro 10.000, 13083-970, Campinas-SP, Brazil - E-mail: douglas.galante@lnls.br.

[5]Instituto de Física, Universidade de São Paulo - Rua do Matão 1371, 05508090, Cidade Universitária, São Paulo-SP, Brazil - E-mail: rizzutto@if.usp.br.

[6] Departamento de Geologia, Universidade Federal de Ouro Preto – Morro do Cruzeiro s/n, 35400-000, Campus Morro do Cruzeiro, Ouro Preto-MG, Brazil - E-mail: idrgeo@gmail.com.

[7]Programa de Pós-Graduação em Química, Instituto de Química, Universidade de São Paulo – Avenida Prof. Lineu Prestes 748, 05508080, Cidade Universitária, São Paulo-SP, Brazil - E-mail: evandro.pereira.silva@usp.br.

[8]Departamento de Química Fundamental, Instituto de Química, Universidade de São Paulo – Avenida Prof. Lineu Prestes 748, 05508080, Cidade Universitária, São Paulo-SP, Brazil - E-mail: farod@iq.usp.br.

[9]Laboratório de Plasmas Tecnológicos, Universidade Estadual Paulista – Avenida Três de Março 511, 18087-180, Sorocaba-SP, Brazil - E-mail: elidiane@sorocaba.unesp.br.
[10]Departamento de Geologia, Universidade Federal de Pernambuco - Avenida Acadêmico Hélio Ramos s/n, 50740530, Cidade Universitária, Recife-PE, Brazil - E-mail: psucerquia@gmail.com.
[11]Departamento de Biologia, Universidade Federal de São Carlos – Rodovia João Leme dos Santos, Km 110, 18052780, Sorocaba-SP, Brazil - E-mail: forancelli.ufscar@gmail.com.

Supplementary information

Supplementary Table – List of analyzed samples and thin sections

Samples	Thin sections	Facies (GL - grey limestones; BL - beige limestones)
GP/2E 9666	GP/L 16, GP/L 17	GL
GP/2E 9006	GP/L 18, GP/L 19	GL
GP/2E 7781g	GP/L 20, GP/L 21	BL
GP/1E 9435	GP/L 172	BL
GP/2E 9005		GL
GP/2E 9014		BL
GP/2E 7786f		BL
GP/2E 7913e		BL
GP/2E 7782j		BL
GP/2E 7780e		BL

Geological context: General overview

Located in north-eastern Brazil, the Araripe Basin (Supplementary Figure 1a,b) was formed during continental break-up[1]. The Aptian-Albian Santana Formation[2,3] is the most studied unit in the Araripe Basin, especially owing to its abundant fossil record and its rock commercial value[4]. This formation lies above the Aptian-Albian[2] Barbalha Formation, and below the Mesoalbian Araripina Formation. In addition, the Santana Formation is divided into two subunits: in the bottom, the Late Aptian Crato Member (with the Ipubi Layers at top), and in the top, the Late Aptian-Early Albian Romualdo Member[1,2]. Additionally, exceptional preservation occurs in both members, conferring them a status of Konservat- and Konzentrat-Lagerstätten[5,6,7], and references therein.

The Crato Member (Supplementary Figure 1c), wherein the studied fossils were unearthed, is composed of six carbonate lacustrine beds (clay-carbonate rhythmites overlaid by laminated limestones) interbedded with deltaic shales, siltstones, and sandstones[1,8]. Evidence for preserved microfossils, EPS, and organomineralization

suggest that the Crato carbonates were precipitated by microbial activity[9], although precipitation triggered by whitings cannot be ruled out[10]. Carbonate facies, which have varied organic matter contents, were deposited in a restricted basin under low energy in anoxic or dysoxic bottom water conditions, as evidenced by well-preserved palynomorphs, amorphous organic matter preservation and iron oxide-hydroxides after pyrite[9,10]. In addition, the analysis of the limestone facies suggests six transgressive-regressive cycles, controlled mainly by climate cycles (humid and dry conditions) and tectonics[8,9]. Located above the Crato Member, the Ipubi Layers record evidence for a transitional setting (evaporitic/lacustrine to lagoon/marine), where thick (ca. 30 m) beds of gypsum and anhydrite occur and are overlaid by shales and sandstones[1,11].

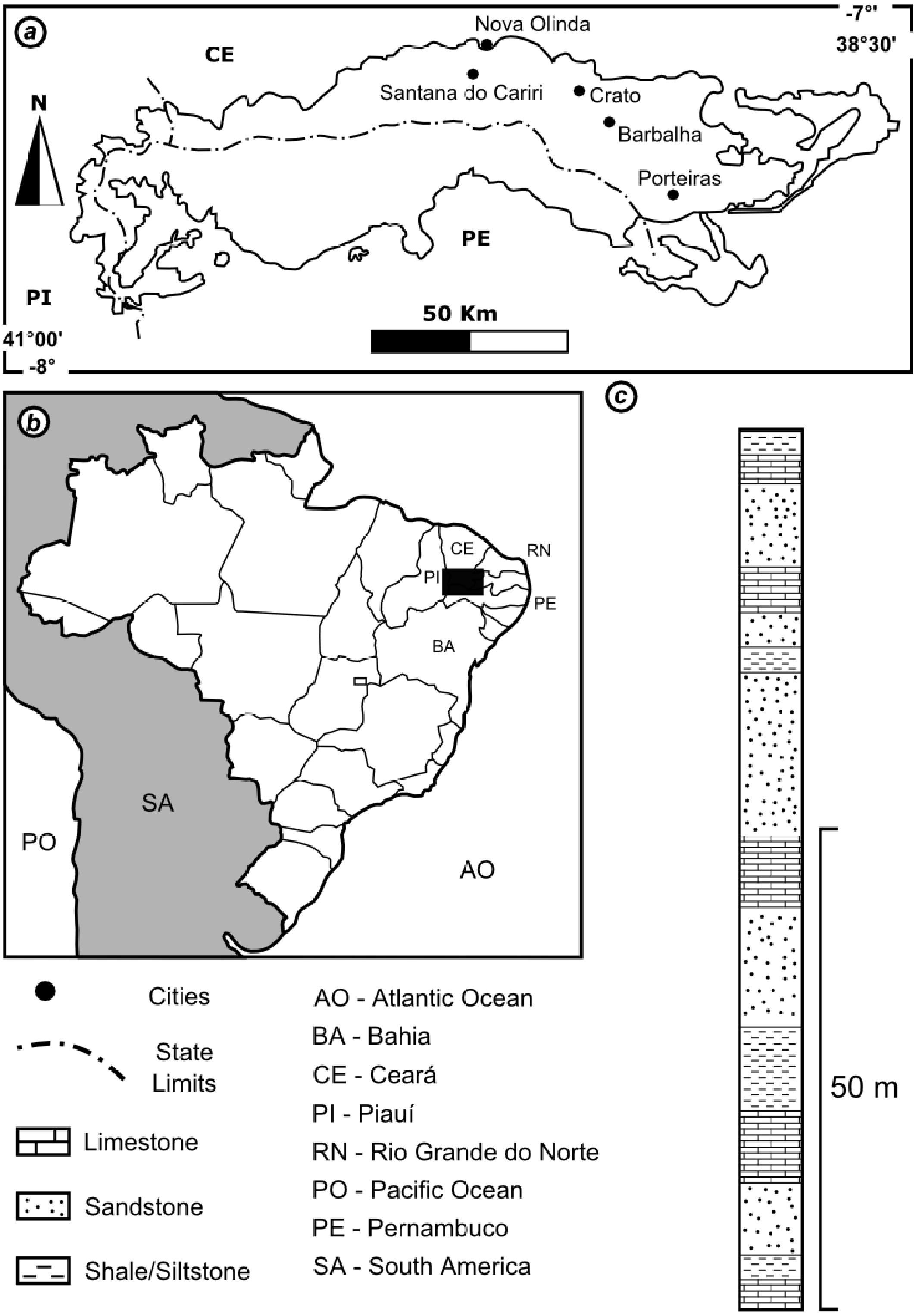

Supplementary Figure 1. Geologic context of the studied fossils. a, The Araripe Basin. **b**, Localization of the Araripe Basin in Brazil. **c**, Representative lithostratigraphic column of the Crato Member. The maps in **a** and **b** were created in the software Inkscape 0.91 (available in <https://inkscape.org/en/>).

Supplementary note 1 – General overview of *Dastilbe crandalli*

Dastilbe crandalli (Gonorynchiformes: Chanidae) is the most common fish from the Crato Member. Its standard length is ca. 100 mm, although specimens up to 210 mm are also found. *D. crandalli* also occurs in the following Brazilian north-eastern sedimentary basins: Sergipe-Alagoas, Parnaíba, São Francisco, Tucano, Pernambuco-Paraíba, besides in Rio Benito, Equatorial Guinea[12]. A Middle Cretaceous vicariance involving the Chanidae clade, particularly between South America (*Tharrhias, Dastilbe*) and Africa (*Parachanos*) has been detected[13]. Freshwater fish dispersal was a common event since several marine barriers were still very narrow and probably subject to width variations due to sea level fluctuations[13]. Although several works deal with *Dastilbe* morphology and palaeogeographic distribution, very few use this fish as a taphonomic tool. In this way, the understanding of labile-tissue preservation sheds light on palaeoenvironmental aspects of *Dastilbe* habitats.

Supplementary note 2 - Comments on integument preservation

While Crato Member pyritized fish have rarely preserved integument, kerogenized specimens commonly display scales interlayered with skin remains. In the case of pyritization, the integument was the first massive source for microbial decay (by sulphate-reducing (SR) bacteria), yielding high sulphide concentrations, even if considering integument to be less decay-prone than muscles. That very high sulphate reduction rates only prompt pyrite coatings around carcasses and soft-tissues[19] accounts for the red halo that usually surrounds fossils as well as the lack of integument preserved in beige limestone (BL) fish. Labile-tissue pyrite coating is uneventful among Konservat-Lagerstätten, being recorded in Beecher's Trilobite Bed and Hunsrück Slate[20]. However, Crato Member fish muscles and sometimes eyes have been preserved in 3D, implying that SR has not been widespread (i.e. SR rates have not been exceedingly high) and pyritization was more concentrated in some tissues. In this way, integument preservation in kerogenized fish is coherent with methanogen

degradation of C_{org} since methanogenesis yields less degradation than SR[16]. Additionally, the lack of skin preservation in BL specimens could be a consequence of *post mortem* rupture near head base, as seen in several specimens (Supplementary Figures 3, 4, and 6). Gas escape, as reported in Romualdo Member (Santana Formation) fish[17], is not likely an explanation for the case of *Dastilbe crandalli*. Taphonomic experiments on lacustrine fish (Series Otophysi, related to Series Anotophysi, in which *D. crandalli* is classified) have shown that gases produced after decay escape from original openings[18]. Therefore, the head-neck rupture described above can be considered a case of tetany (i.e. severe muscular contraction), regarding that rupture was not likely caused by transport[18]. Tetany is caused by (1) thermic abrupt variation, (2) salinity/alkalinity, or (3) anoxia. Based on the fact that among these three possibilities, the second results in body deformation, we considered that salinity is the most plausible cause for head-neck rupture in *D. crandalli* specimens. This hypothesis is in agreement with hypersalinity episodes in the Crato palaeolake[6].

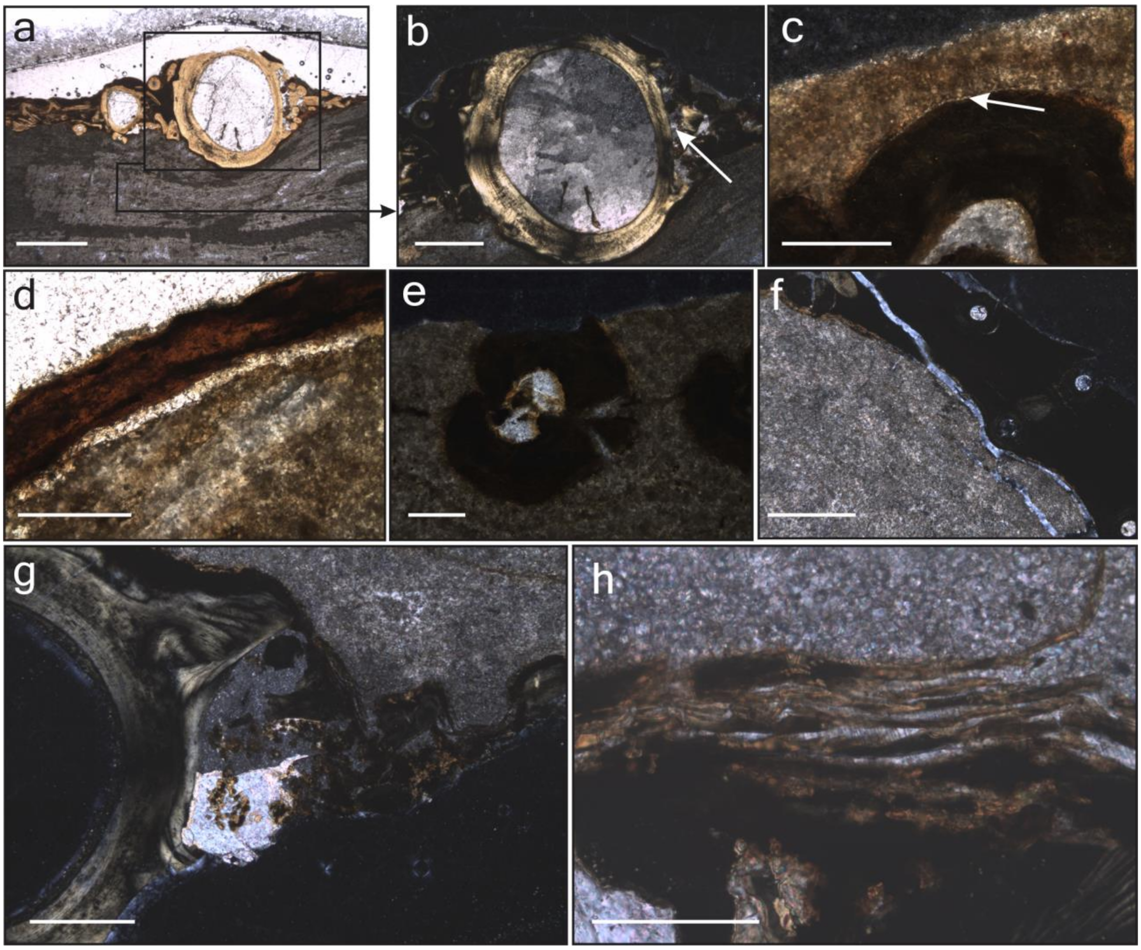

Supplementary Figure 2. Cement types in carbonaceous fish. Thin sections GP/L 16 (a-e) and GP/L 17 (f-h). **a**, Microfault in right half. **b**, Equigranular sparry calcite in mosaic filling the vertebrae, and poikilotopic sparry calcite filling tissue empty spaces around vertebra (arrow). **c**, Sparry calcite outlining bones (arrow). **d**, Sparry calcite outlining soft-tissues. **e**, Sparry calcite filled bones and their fractures. **f**, Apatite veins sometimes outline soft-tissue outer margin (lower half) and cut it inward (upper half). **g**, Poikilotopic sparry calcite around vertebra. **h**, Sparry calcite between scales. Scale bars: **a** – 2 mm; **b** – 1 mm; **c**-**e** – 0.2 mm; **f**, **g** – 0.5 mm; **h** – 0.1 mm.

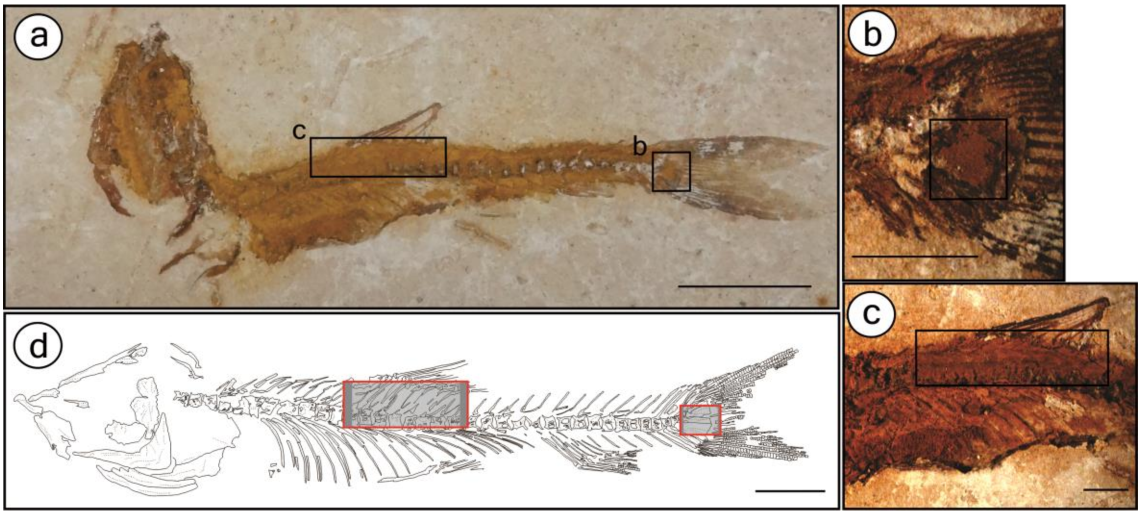

Supplementary Figure 3. Localization of soft-tissues analyzed by SEM. a, Specimen GP/2E 7786f. **b, c,** Regions imaged by SEM are highlighted by rectangles. **d,** General scheme depicting regions marked in **a**. Scale bars: **a** – 10 mm; **b** – 2 mm; **c** – 2 mm; **d** – 10 mm.

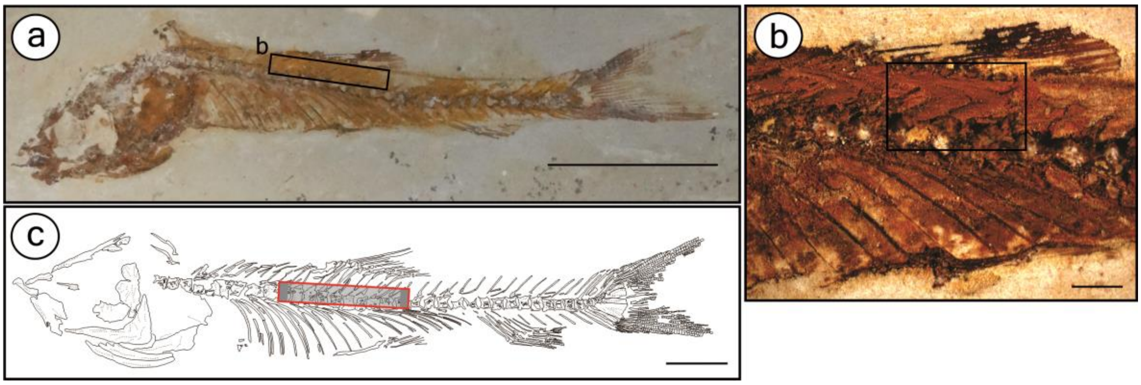

Supplementary Figure 4. Localization of soft-tissues analyzed by SEM. a, Fossil GP/2E 7782j. **b**, Myomeres below dorsal fin (at top). Area analyzed by SEM is highlighted by the rectangle. **c**, General scheme depicting area marked in **a**. Scale bars: **a** – 10 mm; **b** – 1 mm; **c** – 10 mm.

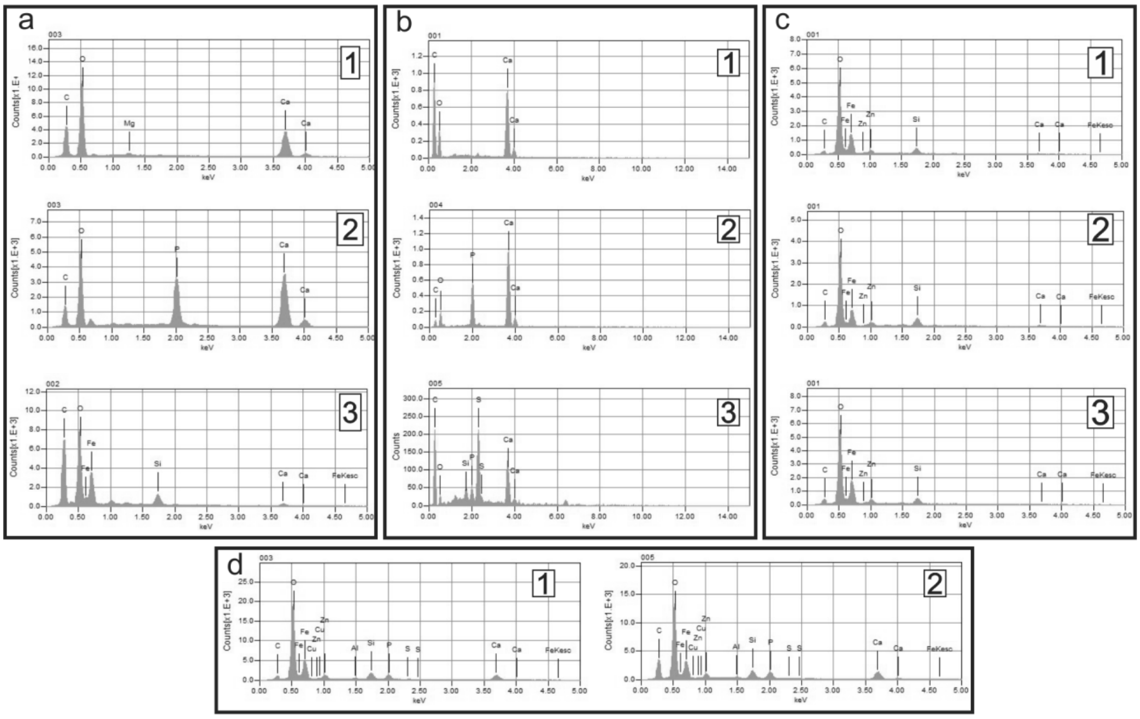

Supplementary Figure 5. EDS point spectra. Thin sections GP/L 20 (**a**) and GP/L 16 (**b**) and specimens GP/2E 7786f (**c**) and GP/2E 7913e (**d**). **a**, 1 – calcite cement, 2 – bone, 3 – soft-tissue. **b**, 1 – calcite cement, 2 – bone, 3 – soft-tissue. **c**, Muscle microfabric composition: 1 – base of caudal fin, 2 – base of dorsal fin, 3 – muscles attached to vertebral column. **d**, 1 – microfabric, 2 – putative EPS.

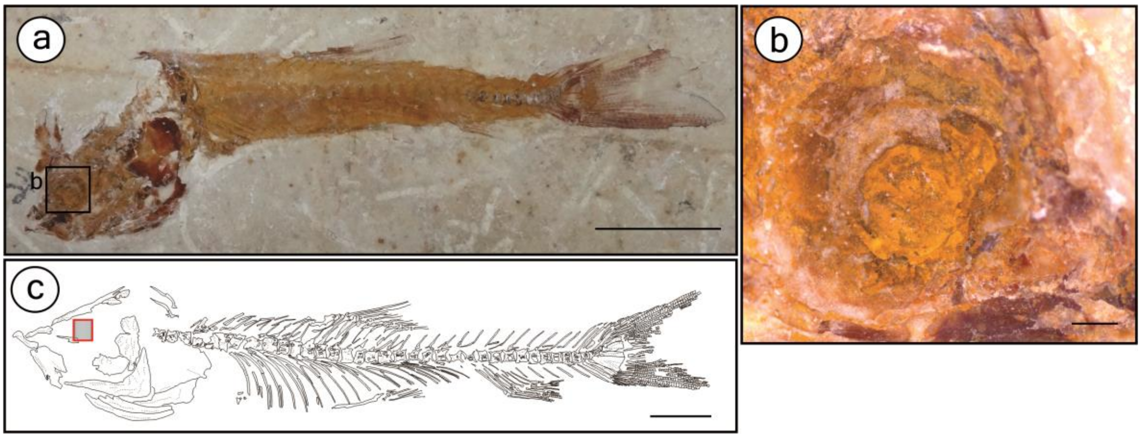

Supplementary Figure 6. Localization of soft-tissues analyzed by SEM. a, Fossil GP/2E 7780e. **b**, Detail of the eye analyzed by SEM. **c**, General scheme depicting area marked in **a**. Scale bars: **a** – 10 mm; **b** – 0.5 mm; **c** – 10 mm.

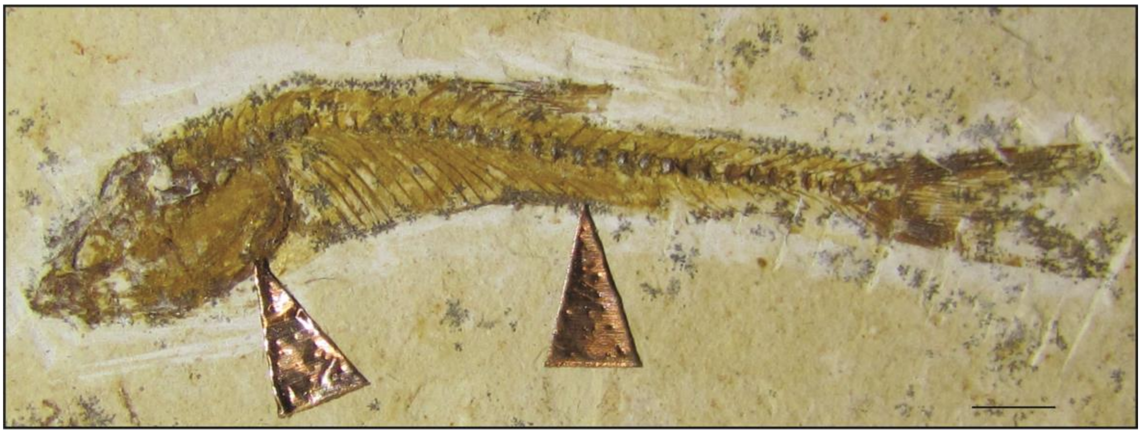

Supplementary Figure 7. Specimen GP/2E 7913e. Scale bar is 3 mm.

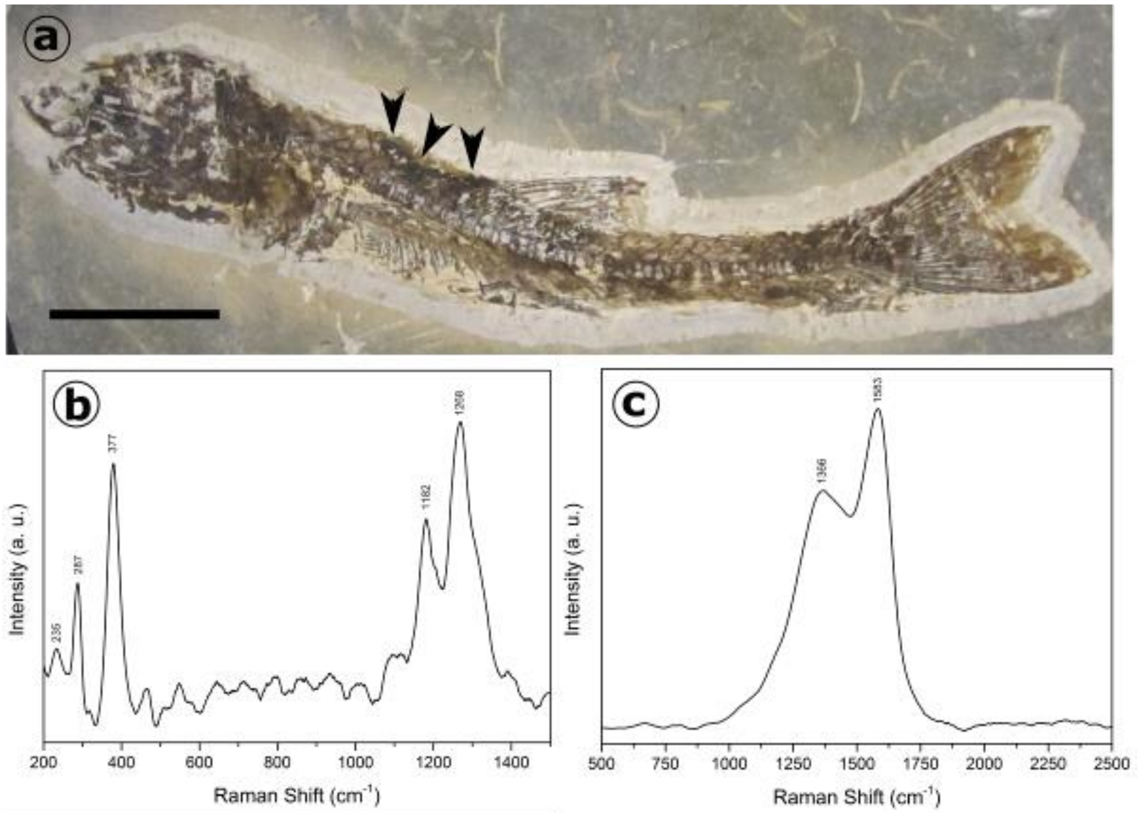

Supplementary Figure 8. Micro-Raman spectra of soft-tissues. a, Specimen GP/2E 9005. Soft-tissues are indicated by arrow heads. Scale bar is 30 mm. **b**, Spectrum of goethite (peaks at 235, 287, and 377 cm-1) of BL fish GP/2E 7786f (Supplementary Figure 3). **c**, Spectrum of kerogen (D band – ca. 1366 cm^{-1}; G band – ca. 1583 cm^{-1}) of fossil in **a**. Intense bands (D and G bands) plus the lack of shoulder associated with the G band suggest that the analyzed material is both highly disordered and poorly geochemical mature, maybe implying that original composition has been little altered[19,20].

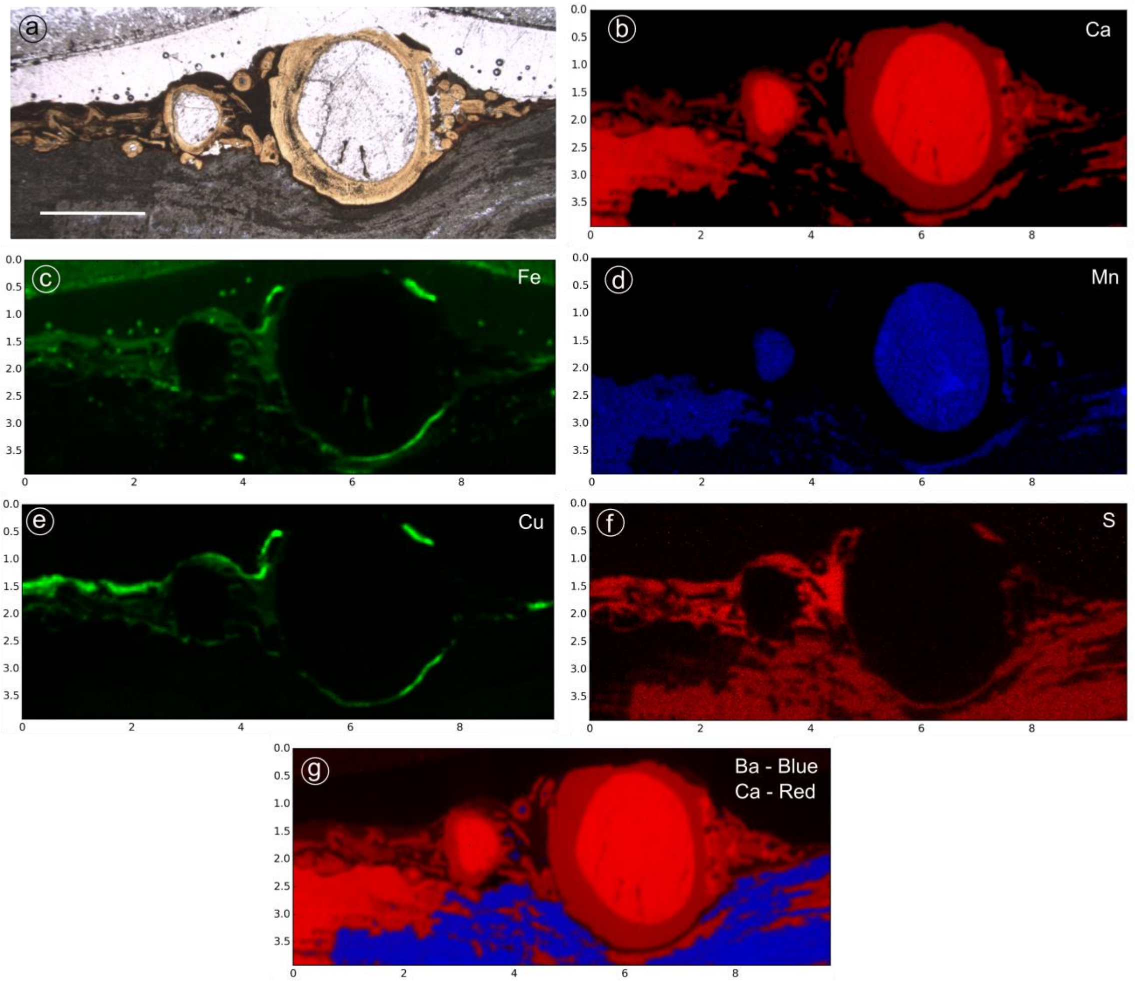

Supplementary Figure 9. SR-µXRF maps of fish (GP/2E 9666) with preserved carbonaceous soft-tissues. a, Area mapped by SR-µXRF of the thin section GP/L 16. **b-g**, SR-µXRF maps. Image brightness is proportional to element concentration, and both map horizontal and vertical scale axes are in mm. Scale bar: **a** – 2mm.

Supplementary References

1. Assine M. L. Bacia do Araripe. *Bol. Geoc. Petr*. **15,** 371-389 (2007).

2. Coimbra, J. C., Arai, M. & Carreño, A. L. Biostratigraphy of lower Cretaceous microfossils from the Araripe Basin, northeastern Brazil. *Geobios* **35,** 687-698 (2002).

3. Rios-Netto, A. D. M., Regali, M. D. S. P., Carvalho, I. D. S. & Freitas, F. I. D. Palinoestratigrafia do intervalo Alagoas da Bacia do Araripe, Nordeste do Brasil. *Rev. Bras. Geoc.* **42,** 331-342 (2002).

4. Campos, D. A., Vidal, F. W. H. & Castro, N. F. *Quarrying limestones and saving fossils of the Araripe Basin, Brazil* (Pacini Editore, Pisa, 2008).

5. Maisey, J. G. *Santana fossils: an illustrated atlas* (T.F.H. Publications, Neptune City, 1991).

6. Martill, D. M., Bechly, G. & Loveridge, R. *The Crato Fossil Beds of Brazil: Window To An Ancient World* (Cambridge University Press, New York, 2007).

7. Barling, N., Martill, D. M., Heads, S.W. & Gallien, F. High fidelity preservation of fossil insects from the Crato Formation (lower Cretaceous) of Brazil. *Cret Res*. **52,** 605-622 (2015).

8. Neumann, V. H. M. L. *Estratigrafia, Sedimentologia, Geoquimica y Diagenesis de los Sistemas Lacustres Aptiense-Albienses de la Cuenca de Araripe (Noreste De Brasil)* (Tesis de Doctorado, Universitat de Barcelona, Barcelona, 1999).

9. Catto, B., Jahnert, R. J., Warren, L. V., Varejao, F. G., Assine, M. L. The microbial nature of laminated limestones: lessons from the Upper Aptian, Araripe Basin, Brazil. *Sediment. Geol.* doi: 10.1016/j.sedgeo.2016.05.007 (2016).

10. Heimhofer, U. et al. Deciphering the depositional environment of the laminated Crato fossil beds (early Cretaceous, Araripe Basin, north-eastern Brazil). *Sedimentology* **57,** 677-694 (2010).

11. Silva, M. A. M. Evaporitos do Cretáceo da Bacia do Araripe: ambientes de deposição e história diagenética. *Bol. Geoc. Petr.* **2,** 53-63 (1988).

12. Brito, P. M., Amaral, C. R. L. *An overview of the specific problems of Dastilbe JORDAN, 1910 (Gonorynchiformes: Chanidae) from the Lower Cretaceous of western Gondwana* (Verlag, Munich, 2008).

13. Cavin, L. Palaeobiogeography of Cretaceous bony fishes (Actinistia, Dipnoi and Actinopterygii). *Geol. Soc. Spec. Publ.* **295,** 165-183 (2008).

14. Canfield, D. E. & Raiswell, R. *Pyrite Formation and Fossil Preservation Ch 7* (Plenum Press, New York, 1991).

15. Allison, P. A. Konservat-Lagerstatten: cause and classification. *Paleobiology* **14,** 331-344 (1988).

16. Berner, R. A. A new geochemical classification of sedimentary environments. *J. Sediment. Petrol.* 51, 359-365 (1981).

17. Martill, D. M. Preservation of fish in the Cretaceous Santana Formation of Brazil. *Palaeontology* **31,** 1-18 (1988).

18. Elder, R. L. *Principles of aquatic taphonomy with examples from the fossil record* (Thesis, University of Michigan, Ann Arbor, 1985).

19. Pasteris, J. D. & Wopenka B. Necessary, but not sufficient: Raman identification of disordered carbon as a signature of ancient life. *Astrobiology* **3,** 727-738 (2003).

20. Schopf, J. W., Kudryavtsev, A. B. Confocal laser scanning microscopy and Raman imagery of ancient microscopic fossils. *Prec. Res.* **173,** 39-49 (2009).
